# Identification and characterization of histone modification gene family reveal their critical responses to flower induction in apple

**DOI:** 10.1186/s12870-018-1388-0

**Published:** 2018-08-20

**Authors:** Sheng Fan, Jue Wang, Chao Lei, Cai Gao, Yang Yang, Youmei Li, Na An, Dong Zhang, Mingyu Han

**Affiliations:** 1 College of Horticulture, Northwest A&F University, Yangling, 712100 Shaanxi China; 2 Innovation Experimental College, Northwest A&F University, Yangling, 712100 Shaanxi China

**Keywords:** *Malus domestica*, Histone modification, Flower induction, Evolution, Expression profile

## Abstract

**Background:**

Histone methylation and acetylation regulate biological processes in plants through various histone modifications (*HMs*) gene families. However, knowledge of *HMs* genes is limited in horticultural deciduous trees, including apple (*Malus domestica*).

**Results:**

Here, a comprehensive study of identifying and investigating *HMs* genes was performed using the recently published apple genome. In total, 198 *MdHMs* were identified, including 71 histone methyltransferases, 44 histone demethylases, 57 histone acetylases, and 26 histone deacetylases. Detailed analysis of the *MdHMs*, including chromosomes locations, gene structures, protein motif and protein-protein interactions were performed, and their orthologous genes were also predicted against nine plant species. Meanwhile, a syntenic analysis revealed that tandem, segmental, and whole genome duplications were involved in the evolution and expansion of the *MdHMs* gene family. Most *MdHMs* underwent purifying selection. The expression profiles of 198 *MdHMs* were investigated in response to 6-BA treatment and different flowering varieties (easy-flowering ‘Yanfu No.6’ and difficult-flowering ‘Nagafu No.2’) using transcriptome sequencing data, and most *MdHMs* were involved in flower induction processes. Subsequent quantitative real-time PCR was then performed to confirm the expression levels of candidate *MdHM*s under different flowering-related circumstances.

**Conclusion:**

*MdHM*s were involved in, and responsive to, flower induction in apple. This study established an *MdHMs* platform that provided valuable information and presented enriched biological theories on flower induction in apple. The data could also be used to study the evolutionary history and functional prospects of *MdHMs* genes, as well as other trees.

**Electronic supplementary material:**

The online version of this article (10.1186/s12870-018-1388-0) contains supplementary material, which is available to authorized users.

## Background

Histone modifications (HMs), which repressed or promoted gene expression, affected various processes and played important roles during plant growth and development. Methylation, demethylation, acetylation and deacetylation were common histone modifications processes. These modifications depended on four different *HMs* gene family members, including histone methyltransferases (*HMTs*), histone demethylases (*HDMs*), histone acetylases (*HATs*), and histone deacetylases (*HDACs*). Similarly, *HMTs*, *HDMs*, *HATs* and *HDACs* regulated various biological processes in plants [1, 2].

These four gene family contained different subfamilies. *HMTs* family included two subfamilies, and they were SDG (set domain group) and PRMT (protein arginine methyltransferases). *HDMs* family also included two subfamilies, HDMA (SWIRM and C-terminal domain) and JMJ (JmjC domain-containing proteins). As for *HATs* family, four kinds of subfamilies (HAG, HAM, HAC and HAF) were contained. I): HAG types included GCN5-, ELP3-, and HAT1-like histone acetylases domain structure; II): HAM types included a MOZ-YBF2 domain; III): HAC types included a p300/CREB-binding protein structure; IV): HAF types included a TATA binding protein-associated factors TAF_II_250. HDACs family shared three subfamilies, including HAD (RPD3/HDA1 superfamily), SRT (silent information regulator 2) and HDT (HD2 families) [3–5]. Totally, each subfamily contained typical domain or structure.

Apart from their different structures, the number of *HMs* genes was also different in plants. A total of 136 *HMs* (47 *HMT*_*S*_, 23 *HDMs*, 50 *HAT*, and 16 *HDACs*) have been identified in sweet orange, and they played important roles in fruit development [6]. Additionally, 125 *HMs* (32 *HATs*, 15 *HDACs*, 52 *HMTs* and 26 *HDMs*) were also identified from tomato genome [7]. In total, 35 *SDGs* members have been identified in the grape genome and some were up-regulated during grape softening [3]. Meanwhile, *HMs* gene functions were partially characterized, especially in the model plant *Arabidopsis*. They played important roles in plant growth and development, including in photomorphogenesis, seed germination and dormancy, embryo development, flowering-related processes, fruit development, stress and defense, and hormonal signaling [8–15]. *HMs* can directly function in regulating flowering through their over expression or down expression. They can also affect the expression of flowering related genes. For example, an *Arabidopsis thaliana HDA* family member, *AtHDA9* (AT3G44680), repressed flowering by affecting the acetylation of *AGAMOUS-LIKE 19* (*AGL19*) [16]. Additionally, *AtHDA19* (AT4G38130) influenced flower development together with A-class organ identity gene *AP2* (*APETALA2*), similar as *AtHDA6* (AT5G63110), which showed late flowering in the *HDA6*-RNAi plants [17, 18]. Other genes, such as *AtHAM1* (At5g64610), *AtHAM2* (At5g09740), *AtHAC1* (At1g79000) were also responsible for flowering time [19–21]. For example, the artificial microRNA *AtHAM1* and *AtHAM2* showed earlier flowering time, while overexpression *AtHAM1* flowered later and had more rosette leaves [20]. In tomato (*Solanum lycopersicum*), *SlHAG22*, *SlHAG8* and *SlHAG18* were involved in vegetative or reproductive development, and *SlSRT2* participated in flowering [7]. Additionally, *HM* genes can also regulate the expression level of flowering-related genes, such as *FLOWERING LOCUS C* (*FLC*), *LEAFY* (*LFY*), *MADS AFFECTING FLOWERING4* (*MAF4*) and (*MAF5*) [20, 22, 23]. For example, the over-expression of *HAM1* resulted in a higher H4 hyperacetylation and H4K5ac at *FLC* in *Arabidopsis* [20]. In *Arabidopsis*, an enriched level of histone H3 acetylation and H3K4 trimethylation at *FLC* and *MAFs* occurred in the histone deacetylase6 mutant (*had6*) [23, 24]. Meanwhile, *FLOWERING LOCUS T* (*FT*) was also influenced by *HMs*. The *Arabidopsis* JmjC family protein T-DNA insertion mutant lines (*atjmj4*, AT4G2040), showed earlier flowering, which might enrich *FT* mRNA and H3K4me3 levels within *FT* chromatin [25]. Among various flowering related genes, *FLC* and *FT* were the main well researched genes that associated with *HMs* [25–27]. These indicated that *HMs* affect or interact with their downstream or upstream flowering genes to control flowering.

Apple (*Malus domestica*) is an economically important fruit tree in temperate regions worldwide, and flower induction is an important issue, which restricted fruit yield and economic incomes [28–30]. Hormones mediated flower induction, with GA (gibberellin) inhibiting flowering and 6BA (6-benzylaminopurine) or sugar promoting flowering, has been characterized and researched in apple [31–33]. Additionally, some important gene families, including *INDETERMINATE DOMAIN* (*IDD*), *SQUAMOSA PROMOTER BINDING PROTEIN-LIKE* (*SPL*), *MADs-box*, and *GIBBERELLIC ACID STIMULATED ARABIDOPSIS* (*GASA*), have also been well identified and reported to regulate flower induction in apple [33–37]. However, less is known about of *HMs* and their potential involvement in apple flower induction. In 2017, with the publication of a new apple genome [38], it is able for us to systematically identify *HMs* gene family in apple and help us to make a comprehensive investigation about their characterizations and potential response to flower induction.

In this study, we identified 198 *HMs* gene members in the apple genome. They were 71 *MdHMTs* (64 *MdSDGs* and 7 *MdPRMTs*), 44 *MdHDMs* (16 *MdHDMAs* and 28 *MdJMJs*), 57 *HATs* (50 *MdHAGs*, 2 *MdHAMs*, 4 *MdHACs*, and 1 *MdHAF*) and 26 *MdHDACs* (16 *MdHDAs*, 3*MdSRTs* and 7 *MdHDTs*). Additionally, their chromosomes locations, gene and protein structures, gene phylogeny, synteny analysis and protein-protein interaction network were also performed. Meanwhile, transcriptomic sequencing of 6BA treated trees and different flowering varieties (Nagafu No.2 and Yanfu No.6) were performed to investigate their potential involvement during apple flower induction. Furthermore, quantitative real-time PCR (qRT-PCR) was employed to investigate the expression levels of candidate *MdHMs* in different tissues (stem, leaf, flower, fruit and bud) and different flowering circumstances (alternate bearing and sugar-treated trees), and various hormones (GA_3_, SA, ABA and MeJA) stress treatment. The results revealed valuable information of *HMs* genes in apple, which might be applicable to other fruit trees.

## Methods

### Identification and chromosomes location of *HMs* gene family in apple

To identify *HMs* gene family members in the apple genome, a HMM file of each domain was obtained from Pfam database (http://pfam.sanger.ac.uk/), as previous studies [6, 7]. These files were then used as a query to search against the apple genome (GDDH13 V1.1) with HMMER3.0 [39]. The detailed accession number of each file was listed as Additional file 1: Table S1. However, there was no available HDT in the Pfam database. Thus, the protein sequences encoded by four *Arabidopsis* HDT genes, *AtHDT1* (At3g44750), *AtHDT2* (At5g22650), *AtHDT3* (At5g03740), and *AtHDT4* (At2g27840), were downloaded from the TAIR database (The Arabidopsis Information Resource; http://www.arabidopsis.org/) and used as a query to search against the Genome Database for Rosaceae [apple genome (GDDH13 V1.1; https://www.rosaceae.org/] to predict candidate *MdHDTs* family members. Finally, the putative *HMs* genes, including *HMTs* (*SDGs* and *PRMTs*), *HDMs* (*HDMAs* and *JMJs*), *HATs* (*HAGs*, *HAMs*, *HACs*, and *HAFs*) and *HDACs* (*HDAs*, *SRTs* and *HDTs*) were manually checked to confirm their highly conserved segments. The relative locations of *HMs* were obtained from the apple genome [38]. They were then named according to their chromosome orders, as previous study [6].

### Phylogenetic tree construction, gene structure, protein motif and domain, and orthologous genes analysis

For phylogenetic analysis, MEGA 7.0 [40] was used to investigate the phylogenetic interactions of *HMs* between apple and *Arabidopsis*. The *Arabidopsis* and apple HMs protein sequences were aligned by the ClustalW program with default parameters. The multiple sequence alignment generated files were analyzed and then used to build phylogenetic trees with Maximum Likelihood method, pairwise deletion for sequences analysis, and a bootstrap value of 1000 times. For the gene structural analysis, gene models were downloaded from apple genome database (https://iris.angers.inra.fr/gddh13/) [38]. They were then submitted into Gene Structure Display Server (http://gsds.cbi.pku.edu.cn/) for structural analyses [41]. Additionally, the HMs protein sequences were employed to investigate conserved motifs with MEME Suite platform (http://meme-suite.org/), and 10 motifs were found within each gene family. Protein-protein interactions were analyzed with http://string-db.org/. For orthologous genes identification, each pair of gene was used to BLAST with sequences homology more than 60% and e-value less than 1e-20. Gene Ontology (GO) terms analysis were performed with online database (http://www.geneontology.org).

### Tandem duplication and synteny analysis

Circos v. 0.63 (http://circos.ca/) was employed to investigate their tandem duplication and synteny relationships as previous methods [33–37]. Tandem duplication *MdHMs* genes were identified according to their physical locations on individual chromosomes in the apple genome. Adjacent homologous *HMs* genes on the same apple chromosome with no more than one intervening gene were considered tandem duplicates. The Plant Genome Duplication Database (http://chibba.agtec.uga.edu/duplication/) was used to identify synteny blocks between *Arabidopsis* and apple. For orthologous gene pairs, synonymous (Ks) and non-synonymous (Ka) nucleotide substitutions were calculated according to the comparative synteny map between the apple and *Arabidopsis thaliana* genomes, with ClustalX and PAL2NAL programs for protein and coding sequences alignment. They were calculated with DNASP v5.10 software.

## Plant materials and treatment

### Trees with different flowering capabilities

In total, 18 six-year-old apple trees of contrast flowering varieties (Yanfu No.6 and Nagafu No.2), which had been planted at the Apple Demonstration Nursery of Yangling Modern Agriculture Technology Park (108°70′E, 34°52’N), were used in this study. They were all grafted T337/ *Malus robusta* Rehd. Additionally, ‘Yanfu No.6’, a spur mutation of ‘Nagafu No.2’, had a higher flowering rate and greater bud morphological development [34, 35]. Trees were divided into three blocks, with three of each, respectively. Terminal buds were collected from current spurs at 30, 50 and 70 days after full bloom (DAFB) [29, 35]. They were then stored for further use.

### Sugar and hormones treated apple trees

An additional 18 uniform ‘Fuji’/ T337/ *Malus robusta* Rehd were used for sucrose treatment experiments in the same orchard. They were also divided into six blocks. Three of them were sprayed with 15,000 and 20,000 mg L^− 1^ sucrose at 29 and 36 DAFB [32], and the remaining blocks were sprayed with water as control. For 6BA treatment, 18 similar trees were used, and 300 mg L^− 1^ 6BA was performed at 27 and 30 DAFB. They were all sprayed on the whole trees with a low-pressure hand wand sprayer in a clear morning. Samples were collected at 30, 50 and 70 DAFB and stored for further use. Meanwhile, 100 mM GA_3_, 300 μM ABA, 100 μM SA and 50 μM MeJA were treated on 2-year-old ‘Nagafu No.2’ trees, as water as control; leaves were collected at 0, 3, 6, and 12 h for each treatment as previous study [36].

### Alternate bearing trees

Six14-year-old alternate bearing ‘Fuji’ trees were used in Tiandu Town, Fufeng, Baoji, Shaanxi (107°57′ E, 34°28′ N) were sampled. Samples were collected from trees in their ‘ON’ years (with a higher flowering rate) and ‘OFF’ years (with a low flowering rate) in 2014 at 30, 90, and 150 DAFB in the morning. Terminal buds of current spurs were collected from trees in their ‘ON’ or ‘OFF’ years and stored for further use.

### Tissues collection

For the tissue-specific expression analyses, various tissues or organs were collected from ‘Fuji’/T337 *M. robusta* Rehd. Flowers were collected on April 9, 2015 during the full-bloom period. Additionally, stems were collected from new shoots with diameters of 2–3 mm, while mature leaves were collected from the adjacent buds. Fruits with diameters of 2–3 cm were also collected. All samples were immediately frozen in liquid nitrogen and stored at − 80 °C until used in the gene expression analyses.

### RNA extraction and cDNA synthesis

Total RNA was extracted from plant tissue samples using the cetyltrimethyl ammonium bromide method with slight modifications [42]. Briefly, 900 μL extraction buffer (2% cetyltrimethyl ammonium bromide, 2.5% PVP-40, 2 M NaCl, 100 mM Tris-HCl [pH 8.0], 25 mM EDTA [pH 8.0], and 2% b-mercaptoethanol) was preheated at 65 °C and added to 2-mL microcentrifuge tubes just before use. Samples containing 200 mg of bud tissue stored at − 80 °C were ground to a powder, added to the tubes, and mixed with extraction buffer. After shaking and inverting each tube vigorously for 5 min and incubating at 65 °C for 30 min, an equal volume of chloroform:isoamyl alcohol (24:1, *v*/v) was added. Each tube was shaken and inverted vigorously and then centrifuged at 12,000×g for 10 min at 4 °C. For each sample, the supernatant was collected into a new tube and re-extracted with an equal volume of chloroform:isoamyl alcohol (24:1, v/v). The resulting supernatant was then transferred into a new 2-mL tube and LiCl (3 M final concentration) was added. The mixture was incubated at − 20 °C for 4 h and the RNA was selectively pelleted by LiCl after centrifugation at 18,000×g for 20 min at 4 °C. The pellet was resuspended in 500 μL of SSTE buffer (10mMTris-HCl [pH 8.0], 1 mM EDTA [pH 8.0], 1% SDS, and 1 M NaCl) that had been preheated to 65 °C and an equal volume of chloroform:isoamyl alcohol. The mixture was then centrifuged at 12,000×g for 10 min at 4 °C. The supernatant was transferred to a new microcentrifuge tube, and the RNA was precipitated with 2.5 volumes of cold ethanol at − 80 °C for at least 30 min and centrifuged at 1,2000×g for 20 min at 4 °C. Finally, the pellets were washed with 70% ethanol and resuspended in diethylpyrocarbonate-treated water. Total RNA integrity levels were verified by running the samples on 2% agarose gels. First-strand cDNA was synthesized from 1 μg of total RNA using a PrimeScript RT Reagent kit with gDNA Eraser (Takara Bio, Shiga, Japan) following the manufacturer’s instructions.

### Gene expression analysis

The expression levels of the 12 candidate *HM* genes were analyzed using quantitative real-time PCR **(**qRT-PCR). Primers were designed to span an intron-exon junction with Primer Premier 6.0 software. And they were designed with the preferred values to specific amplification with high yield as follows. (1) Length of PCR primers (18–24 bp); (2) Melting temperature (60 °C); (3) GC content (40–60%); (4) GC Clamp: more than 3 G’s or C’s should be avoided in the last 5 bases at the 3′ end of the primer. (5) Avoided hairpins, self and cross dimer, and repeats. (6) Avoid template secondary structure and cross homology. The qRT-PCR mix (20 μL) consisted of 2-μL cDNA samples (diluted 1:8), 10 μL 2× SYBR Premix ExTaq II (Takara Bio), 0.8 μL of each primer (10 μM) (Additional file 2: Table S2), and 6.4 μL distilled deionized H2O. Each PCR assay was run on an iCycler iQ5 Real Time PCR Detection System (Bio-Rad, Plano, TX, USA) with an initial denaturation at 95 °C for 3 min, followed by 40 cycles at 94 °C for 15 s, 62 °C for 20 s, and 72 °C for 20 s. The resulting fragments were subjected to melting-curve analysis to verify the presence of gene-specific PCR products. The melting curve analysis was performed directly after real-time PCR and included an initial step of 94 °C for 15 s, followed by a constant increase from 60 °C to 95 °C at a 2% ramp rate. The apple *EF*-*1*α gene (GenBank accession No. DQ341381) was used as an internal control to normalize all mRNA expression levels under different treatments and different tissues [34–36]. Experiments were performed using three biological replicates with three technical replicates. The 2^−ΔΔCt^ method was used to calculate the relative amount of template present in each PCR amplification mixture [43].

### Statistical analysis

Gene expression data of RT-qPCR were subjected to an analysis of variance (ANOVA) at the 5% level with the SPSS 11.5 software package (SPSS, Chicago, IL, USA). Figures were constructed using Origin 8.0 (Microcal Software Inc., Northampton, MA, USA).

## Results

### Genome-wide identification of *HMs* gene family in the apple genome

**Fig. 1:**
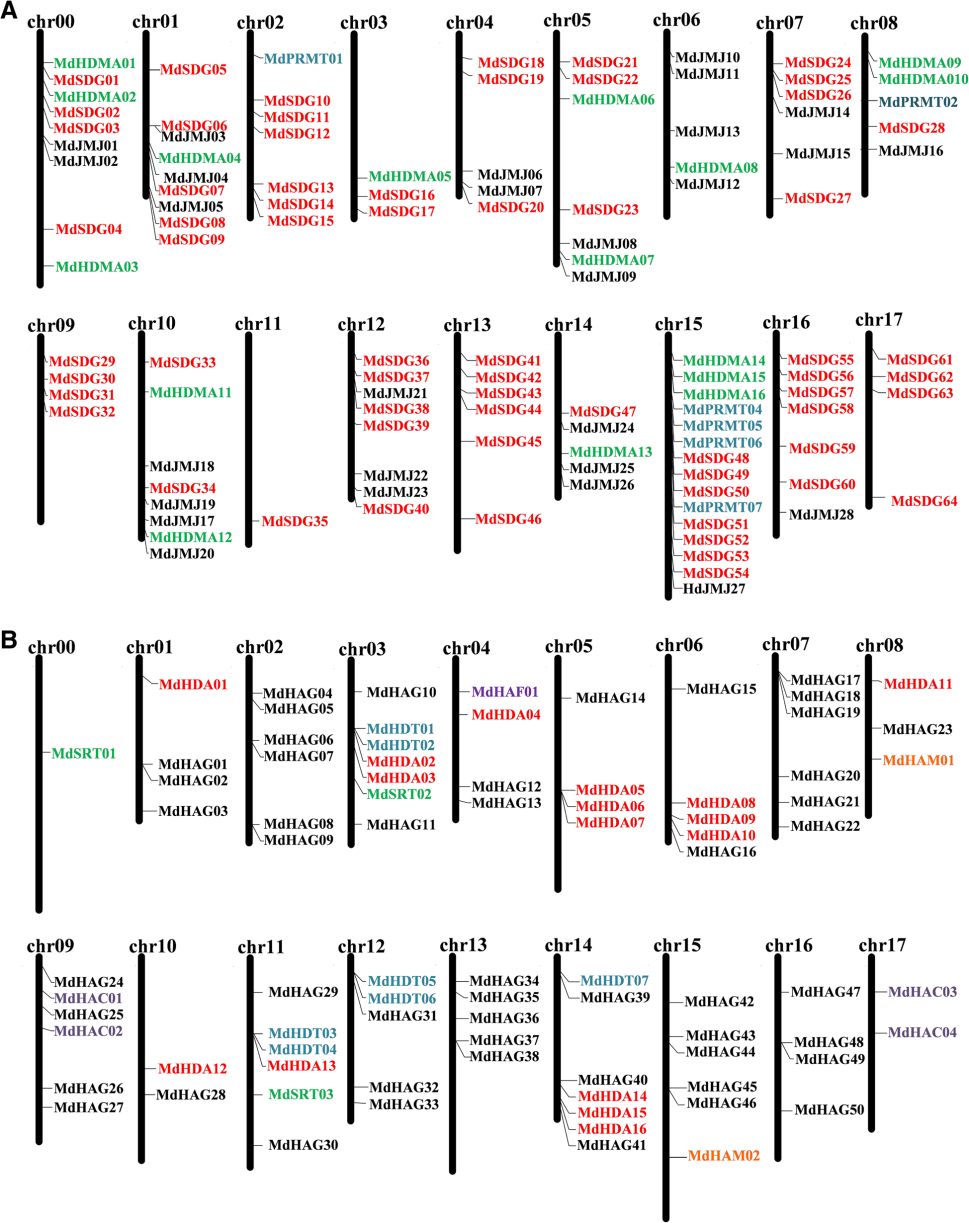
Chromosomes locations of *MdHMs* gene family on the apple genome. **a** Apple histone methyltransferase genes *MdHMTs* (*MdSDGs* and *MdPRMTs*) and histone demethylase genes *MdHDMs* (*MdHMAs* and *MdJMJs*); **b** Apple histone acetyltransferase genes *MdHATs* (*MdHAGs*, *MdHAMs*, *MdHACs*, and *MdHAFs*) and histone deacetylase genes *MdHDACs* (*MdHDAs*, *MdSRTs*, and *MdHDTs*)

**Fig. 2:**
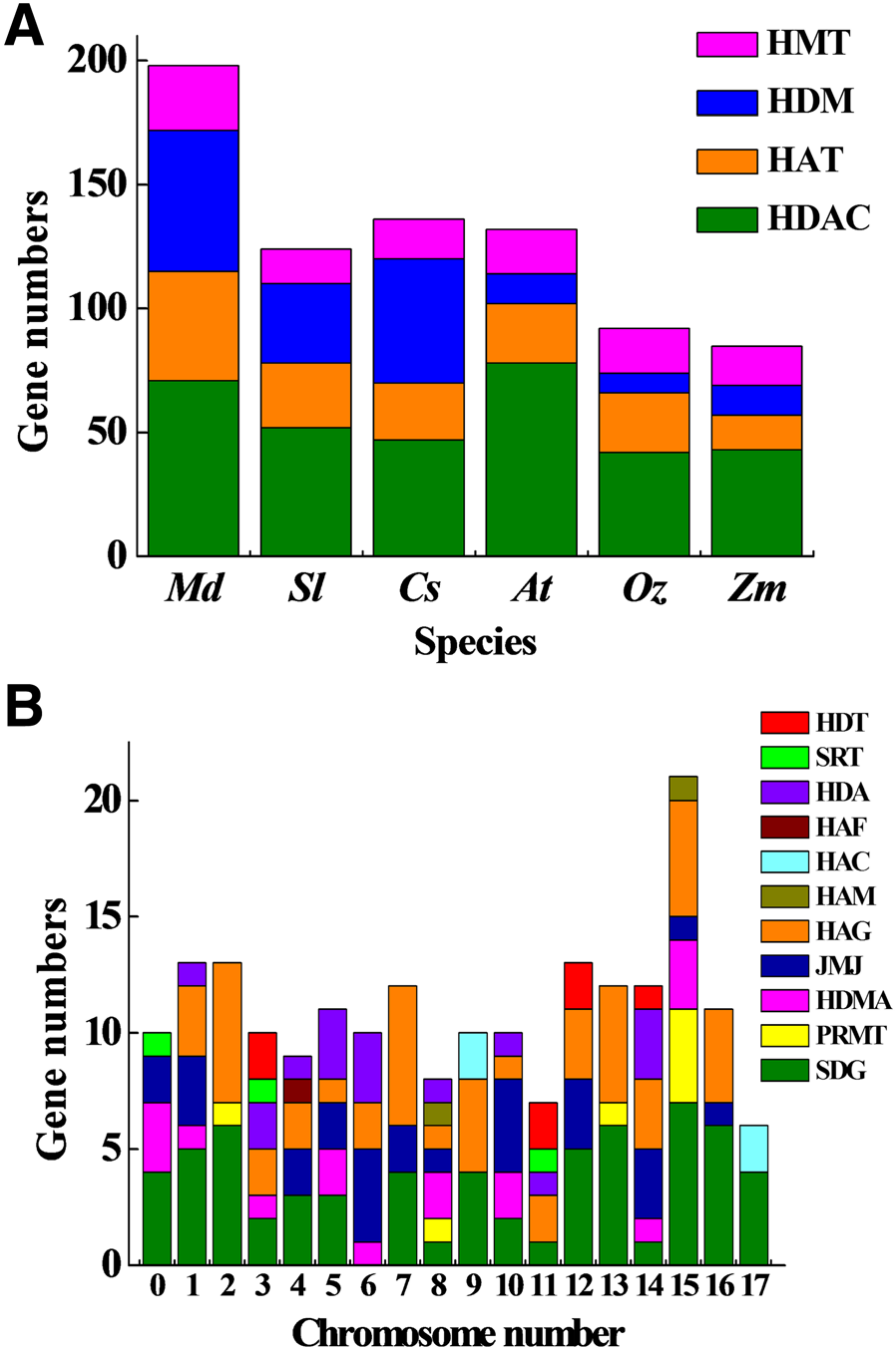
Comparison the number of *HMs* genes among different species and different chromosomes. **a** Analysis of *HMs* members within six different species; *Md*, *Malus domesctia*; *Sl*, *Solanum lycopersicum*; *Cs*, *Citrus sinensis*; *At*, *Arabidopsis thaliana*; *Os*, *Oryza sativa*; *Zm*, *Zea mays.* **b** Comparison of different gene family members on apple genomes; each gene family was present with different color

In total, 198 *HMs* were identified in the apple genome, including 71 *HMTs*, 44 *HDMs*, 57 *HATs* and 26 *HDACs* (Figs. 1a, 2a). All of the *MdHMs* were classified into 11 subfamilies according to their different protein domain. For example, the *HMTs* included 64 *SDGs* and 7 *PRMTs*, the *44 HDMs* included 16 *HDMAs* and 28 *JMJs*, the 57 *HATs* included 50 *HAGs*, 2 *HAMs*, 4 *HACs*, and 1 *HAF* gene, and the 26 *HDACs* included 16 *HADs*, 3 *SRTs*, and 7 *HDTs*. Meanwhile, a detailed GO annotation was provided for all the *HMs* (Additional file 3: Table S3). These *HMs* genes were divided into three categories, including biological process, cellular component and molecular function.

### Chromosome distributed of different *HMs*

To clearly identify the *HM*s, each of the *HMs* were named based on their chromosomal locations (Fig. 1, Table 1), as *MdSDG*s, *MdPRMT*s, *MdHDMA*s, *MdJMJ*s, *MdHAGs*, *MdHAM*s, *MdHAC*s, *MdHAF*s, *MdHDA*s, *MdSRT*s, and *MdHDT*s. All genes were distributed from chromosome 00 to chromosome 17 on the apple genome. Chromosome 15 contained the greatest number of *HMs* (Fig. 2b), followed by chromosome 12. Chromosome 17 had the lowest number of *HMs* genes. The 64 *MdSDGs* genes were distributed on all chromosomes except chromosome 6. Chromosome 15 contained the greatest number of *MdSDGs* genes. Seven *MdPRMTs* genes were distributed on four chromosomes (chromosome 2, 8 13 and 15). Additionally, 16 *MdHDMAs* and 28 *MdJMJs* were widely distributed throughout the apple genome, except on chromosome 2, 11, 13 and 17. The 50 *MdHAGs* were distributed throughout 16 of the 18 chromosomes; however, chromosomes 0 and 17 lacked *MdHAGs* genes. The remaining groups of *HAT*s, including *MdHAM*s, *MdHAC*s, and *MdHAF*s, had partial distributions, similar to those of the *MdHDAC*s.

**Table 1:** List of *MdHMs* gene families in the apple genome

Gene Name	Gene Locus^a^	CDS/bp	Strand
*SDG* gene family			
*MdSDG01*	MD00G1030500	1515	*+*
*MdSDG02*	MD00G1060700	2277	*+*
*MdSDG03*	MD00G1068300	2148	*+*
*MdSDG04*	MD00G1179500	1875	*–*
*MdSDG05*	MD01G1012000	1149	*+*
*MdSDG06*	MD01G1080200	3231	*–*
*MdSDG07*	MD01G1116500	1371	*–*
*MdSDG08*	MD01G1220300	3249	*–*
*MdSDG09*	MD01G1224300	1062	*–*
*MdSDG10*	MD02G1157000	4524	*–*
*MdSDG11*	MD02G1174900	478	*–*
*MdSDG12*	MD02G1195100	1212	*–*
*MdSDG13*	MD02G1265700	2253	*–*
*MdSDG14*	MD02G1267300	2070	*–*
*MdSDG15*	MD02G1278400	3564	*+*
*MdSDG16*	MD03G1258900	891	*–*
*MdSDG17*	MD03G1294100	2451	*+*
*MdSDG18*	MD04G1028500	1623	*–*
*MdSDG19*	MD04G1052400	3861	*+*
*MdSDG20*	MD04G1231900	1740	*–*
*MdSDG21*	MD05G1027900	2019	*+*
*MdSDG22*	MD05G1031300	781	*+*
*MdSDG23*	MD05G1244800	768	*+*
*MdSDG24*	MD07G1051900	2067	*+*
*MdSDG25*	MD07G1058000	1611	*+*
*MdSDG26*	MD07G1058100	1839	*–*
*MdSDG27*	MD07G1289800	3225	*+*
*MdSDG28*	MD08G1159600	6348	*+*
*MdSDG29*	MD09G1002600	2031	*+*
*MdSDG30*	MD09G1103200	7443	*+*
*MdSDG31*	MD09G1105100	1446	*+*
*MdSDG32*	MD09G1129500	1686	*–*
*MdSDG33*	MD10G1032900	3441	*+*
*MdSDG34*	MD10G1226200	1467	*+*
*MdSDG35*	MD11G1279700	3213	*–*
*MdSDG36*	MD12G1009500	1035	*–*
*MdSDG37*	MD12G1043600	429	*–*
*MdSDG38*	MD12G1052100	990	*+*
*MdSDG39*	MD12G1112200	2115	*–*
*MdSDG40*	MD12G1250000	753	*–*
*MdSDG41*	MD13G1020900	1437	*+*
*MdSDG42*	MD13G1069000	1635	*+*
*MdSDG43*	MD13G1130100	2043	*–*
*MdSDG44*	MD13G1134900	2019	*–*
*MdSDG45*	MD13G1224000	1893	*–*
*MdSDG46*	MD13G1279000	2406	*+*
*MdSDG47*	MD14G1101300	1524	*–*
*MdSDG48*	MD15G1130200	6231	*+*
*MdSDG49*	MD15G1133700	6369	*–*
*MdSDG50*	MD15G1141800	1167	*–*
*MdSDG51*	MD15G1271600	4518	*–*
*MdSDG52*	MD15G1285900	2793	*–*
*MdSDG53*	MD15G1338000	1146	*–*
*MdSDG54*	MD15G1356600	1443	*–*
*MdSDG55*	MD16G1019300	1452	*+*
*MdSDG56*	MD16G1067900	1476	*–*
*MdSDG57*	MD16G1130300	2013	*–*
*MdSDG58*	MD16G1130700	1242	*–*
*MdSDG59*	MD16G1228800	2043	*–*
*MdSDG60*	MD16G1258900	1473	*+*
*MdSDG61*	MD17G1006800	2161	*+*
*MdSDG62*	MD17G1091000	7386	*+*
*MdSDG63*	MD17G1118300	1493	*–*
*MdSDG64*	MD17G1287300	1446	*–*
*PRMT* gene family			
*MdPRMT01*	MD02G1037100	1152	*+*
*MdPRMT02*	MD08G1132700	1650	*+*
*MdPRMT03*	MD13G1168500	1638	*–*
*MdPRMT04*	MD15G1111800	726	*–*
*MdPRMT05*	MD15G1112100	1950	*–*
*MdPRMT06*	MD15G1112600	1266	*–*
*MdPRMT07*	MD15G1177300	1149	*+*
*HDMA* gene family			
*MdHDMA01*	MD00G1030000	2382	*+*
*MdHDMA02*	MD00G1041900	2361	*+*
*MdHDMA03*	MD00G1206800	1320	*+*
*MdHDMA04*	MD01G1103000	2712	*–*
*MdHDMA05*	MD03G1220300	2247	*–*
*MdHDMA06*	MD05G1067900	3012	*–*
*MdHDMA07*	MD05G1344100	1446	*–*
*MdHDMA08*	MD06G1137800	1692	*+*
*MdHDMA09*	MD08G1004400	2448	*+*
*MdHDMA10*	MD08G1017300	5703	*+*
*MdHDMA11*	MD10G1077800	3000	*–*
*MdHDMA12*	MD10G1320100	1497	*–*
*MdHDMA13*	MD14G1152600	1638	*+*
*MdHDMA14*	MD15G1003800	2448	*+*
*MdHDMA15*	MD15G1016200	5337	*+*
*MdHDMA16*	MD15G1018300	2355	*+*
*JMJ* gene family			
*MdJMJ01*	MD00G1097500	3129	*–*
*MdJMJ02*	MD00G1097600	564	*–*
*MdJMJ03*	MD01G1082300	3300	*–*
*MdJMJ04*	MD01G1106000	4812	*–*
*MdJMJ05*	MD01G1218500	4560	*+*
*MdJMJ06*	MD04G1202800	1803	*+*
*MdJMJ07*	MD04G1229800	3648	*–*
*MdJMJ08*	MD05G1326700	3075	*–*
*MdJMJ09*	MD05G1351300	2664	*–*
*MdJMJ10*	MD06G1012500	3141	*–*
*MdJMJ11*	MD06G1026100	2673	*+*
*MdJMJ12*	MD06G1159300	4404	*–*
*MdJMJ13*	MD06G1081900	5532	*–*
*MdJMJ14*	MD07G1099600	3702	*–*
*MdJMJ15*	MD07G1172400	4785	*–*
*MdJMJ16*	MD08G1186800	2946	*+*
*MdJMJ17*	MD10G1304800	3093	*–*
*MdJMJ18*	MD10G1182700	2658	*+*
*MdJMJ19*	MD10G1241100	3123	*–*
*MdJMJ20*	MD10G1325700	2070	*–*
*MdJMJ21*	MD12G1046300	1551	*–*
*MdJMJ22*	MD12G1216600	2841	*+*
*MdJMJ23*	MD12G1246900	3711	*–*
*MdJMJ24*	MD14G1103700	5529	*–*
*MdJMJ25*	MD14G1165600	4401	*–*
*MdJMJ26*	MD14G1175900	1425	*+*
*MdJMJ27*	MD15G1372700	2928	*+*
*MdJMJ28*	MD16G1280000	2667	*–*
*HAG* gene family			
*MdHAG01*	MD01G1105800	702	*–*
*MdHAG02*	MD01G1108300	885	*–*
*MdHAG03*	MD01G1237100	855	*+*
*MdHAG04*	MD02G1072700	1425	*–*
*MdHAG05*	MD02G1091000	867	*–*
*MdHAG06*	MD02G1183200	1251	*–*
*MdHAG07*	MD02G1187100	1872	*–*
*MdHAG08*	MD02G1300400	645	*–*
*MdHAG09*	MD02G1300500	663	*–*
*MdHAG10*	MD03G1064100	1173	*+*
*MdHAG11*	MD03G1263400	525	*+*
*MdHAG12*	MD04G1177300	282	*+*
*MdHAG13*	MD04G1217600	507	*+*
*MdHAG14*	MD05G1042000	303	*+*
*MdHAG15*	MD06G1040400	462	*–*
*MdHAG16*	MD06G1234100	810	*–*
*MdHAG17*	MD07G1016800	411	*–*
*MdHAG18*	MD07G1016300	411	*–*
*MdHAG19*	MD07G1023300	615	*+*
*MdHAG20*	MD07G1174400	891	*–*
*MdHAG21*	MD07G1238600	855	*–*
*MdHAG22*	MD07G1309700	852	*+*
*MdHAG23*	MD08G1142000	255	*–*
*MdHAG24*	MD09G1002300	632	*–*
*MdHAG25*	MD09G1120700	600	*–*
*MdHAG26*	MD09G1224900	1231	*+*
*MdHAG27*	MD09G1249800	492	*+*
*MdHAG28*	MD10G1193500	1653	*–*
*MdHAG29*	MD11G1067900	1179	*+*
*MdHAG30*	MD11G1284200	525	*+*
*MdHAG31*	MD12G1036200	297	*+*
*MdHAG32*	MD12G1192400	747	*+*
*MdHAG33*	MD12G1234800	582	*+*
*MdHAG34*	MD13G1053400	579	*+*
*MdHAG35*	MD13G1088000	1092	*+*
*MdHAG36*	MD13G1155300	1173	*+*
*MdHAG37*	MD13G1195500	666	*–*
*MdHAG38*	MD13G1195600	222	*+*
*MdHAG39*	MD14G1023000	462	*+*
*MdHAG40*	MD14G1163700	1695	*+*
*MdHAG41*	MD14G1240800	693	*–*
*MdHAG42*	MD15G1118400	489	*+*
*MdHAG43*	MD15G1202500	1425	*–*
*MdHAG44*	MD15G1217100	1023	*–*
*MdHAG45*	MD15G1295100	1251	*+*
*MdHAG46*	MD15G1298200	1860	*–*
*MdHAG47*	MD16G1088300	1035	*+*
*MdHAG48*	MD16G1196400	459	*+*
*MdHAG49*	MD16G1196500	561	*+*
*MdHAG50*	MD16G1262600	792	*–*
*HAM* gene family			
*MdHAM01*	MD08G1173300	1465	*+*
*MdHAM02*	MD15G1358600	1338	*+*
*HAC* gene family			
*MdHAC01*	MD09G1082800	3288	+
*MdHAC02*	MD09G1170000	5244	–
*MdHAC03*	MD17G1073200	4473	+
*MdHAC04*	MD17G1157200	2616	+
*HAF* gene family			
*MdHAF01*	MD04G1047300	5601	*+*
*HDA* gene family			
*MdHDA01*	MD01G1005700	1338	*–*
*MdHDA02*	MD03G1137300	1389	*+*
*MdHDA03*	MD03G1154900	1149	*–*
*MdHDA04*	MD04G1077800	387	*+*
*MdHDA05*	MD05G1146000	1488	*+*
*MdHDA06*	MD05G1147700	297	*+*
*MdHDA07*	MD05G1149000	1008	*+*
*MdHDA08*	MD06G1167500	1413	*+*
*MdHDA09*	MD06G1202300	2004	*+*
*MdHDA10*	MD06G1211800	1722	*–*
*MdHDA11*	MD08G1043300	1203	*+*
*MdHDA12*	MD10G1145400	1488	*+*
*MdHDA13*	MD11G1159400	1293	*+*
*MdHDA14*	MD14G1173000	1407	*+*
*MdHDA15*	MD14G1211400	2001	*+*
*MdHDA16*	MD14G1222300	1722	*–*
*SRT* gene family			
*MdSRT01*	MD00G1091800	1416	*+*
*MdSRT02*	MD03G1179400	1454	*+*
*MdSRT03*	MD11G1199100	1416	*+*
*HDT* gene family			
*MdHDT01*	MD03G1134300	624	*–*
*MdHDT02*	MD03G1134400	321	*–*
*MdHDT03*	MD11G1156500	594	*–*
*MdHDT04*	MD11G1156600	321	*–*
*MdHDT05*	MD12G1016900	521	*–*
*MdHDT06*	MD12G1017000	315	*–*
*MdHDT07*	MD14G1014900	642	*–*

^a^Gene ID in the apple (*Malus × domestica*) genome (*Malus domestica* Genome GDDH13 Version 1.1);

### Phylogenetic and synteny analysis of *HMs* genes between apple and *Arabidopsis*

**Fig. 3:**
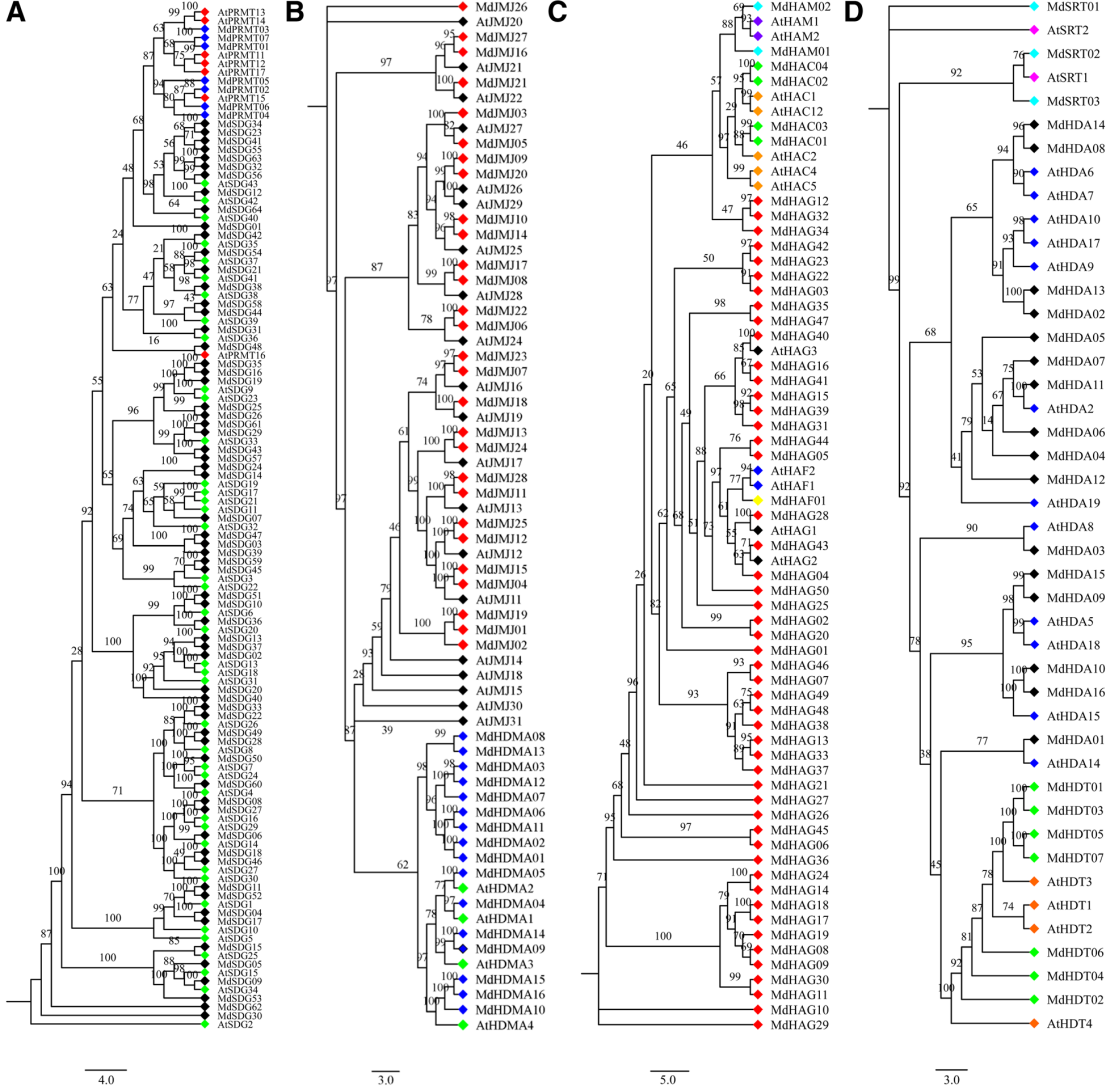
Phylogenetic analysis of *HMs* genes between apple and *Arabidopsis*. **a** *SDG*s and *PRMTs*; **b** *HDMAs* and *JMJs*; **c** *HAGs*, *HAMs*, *HACs*, and *HAFs*; **d** *HDAs*, *SRTs*, and *HDTs*

To understand their evolutionary relationship among *HMs* genes, four rooted phylogenetic trees, including *HMTs*, *HDMs*, *HATs* and *HDACs* genes, were built with *Arabidopsis* and apple HMs proteins (Fig. 3). All *Arabidopsis* and apple HMs genes were classified and clustered into different trends. For *HMTs*, all the *SDGs* and *PRMTs* genes were clustered together, with an exception of *AtPRMT16* (Fig. 3a). Additionally, *HDMAs* and *JMJs* were also clustered with each other (Fig. 3b). For *HATs*, three *HAFs* genes (*AtHAF1*, *AtHAF2*, and *MdHAF01*) were clustered and surrounded by *HAGs* gene members, and other *HAMs* and *HACs* were also tightly grouped with themselves (Fig. 3c)*.* For *HDACs*, three subfamilies (HDAs, HDTs and SRTs) were also clustered (Fig. 3d).

**Fig. 4:**
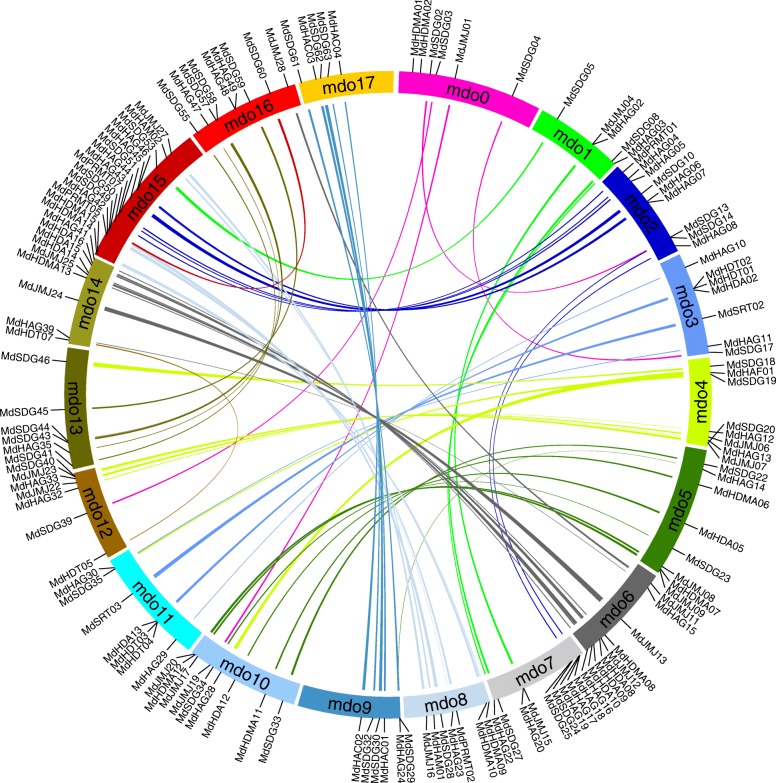
Synteny of *MdHMs* genes in the apple genome. Colored lines with two connected genes within indicated syntenic regions. Detailed information was listed as Additional file 4: Table S4

To characterize the expansion patterns of the *HMs* in the apple genome, a diagram together with Circos algorithm was performed and generated to investigate the duplicated blocks within the apple genome. A total of 67 pairs of *HMs* were identified from 18 chromosomes (Fig. 4, Additional file 4: Table S4), including one pair of *MdHAFs*, *MdSRTs* and *MdHAMs*, two pairs of *MdPRMTs* and *MdHACs*, three pairs of *MdHDTs*, five pairs of *MdHDAs* and *HDMAs*, 10 pairs of *MdJMJs*, 16 pairs of *MdHAGs* and 20 pairs of *MdSDGs*. These paired duplicated genes were all located in different chromosomes, chromosome 15 contained the most *HMs* genes (Fig. 1). Chromosome 1 had the lowest gene number. Totally, these duplicated genomic regions contributed to expansion of *MdHMs* family.

**Fig. 5:**
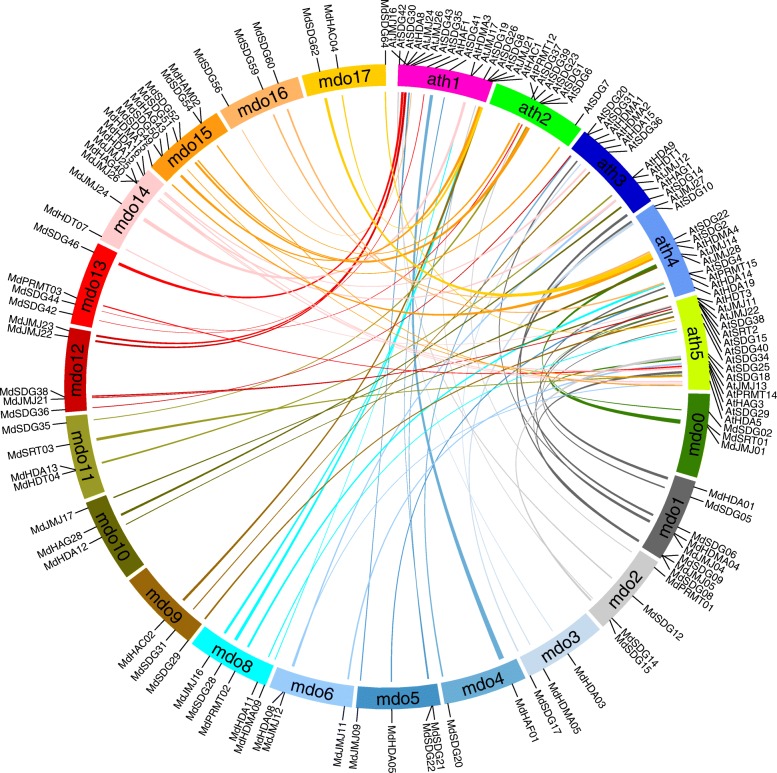
Synteny of *HMs* genes in apple and *Arabidopsis* genome. Colored lines with two connected genes within indicated syntenic regions. Detailed information was listed as Additional file 5: Table S5

Additionally, a syntenic map of *MdHMs* and *AtHMs* were also created to help better understand their potential evolutionary and functional relationships. As shown in (Fig. 5, Additional file 5: Table S5), 72 orthologous pairs of *MdHM*s and *AtHM*s were found in the apple and *Arabidopsis* genome, including two pairs of *HACs*, *SRTs* and *HDTs*, one pair of *HAFs* and *HAMs*, three pairs of *HAGs* and *PRMTs*, 13 pairs of *HDMAs*, 14 pairs of *JMJs* and 31 pairs of *SDGs*. The remaining *HMs* genes did not have ortholog pairs. In addition, to understand the divergence among the orthologous gene pairs of apple and *Arabidopsis*, the ratio of the non-synonymous to the synonymous substitution rate (Ka/Ks) was used to evaluate the selection pressure during duplication. The Ka and Ks value was smaller in apple than betwen *Arabidopsis* and apple. However, the Ka/Ks values between gene pairs in apple less than 1, which was similar to apple and *Arabidopsis* (Additional file 6: Figure S1). The average Ka/Ks ratio between gene pairs in apple was 0.267, which was larger than between gene pairs in apple and *Arabidopsis* (0.106).

### Structure analysis of *MdHMs*

**Fig. 6:**
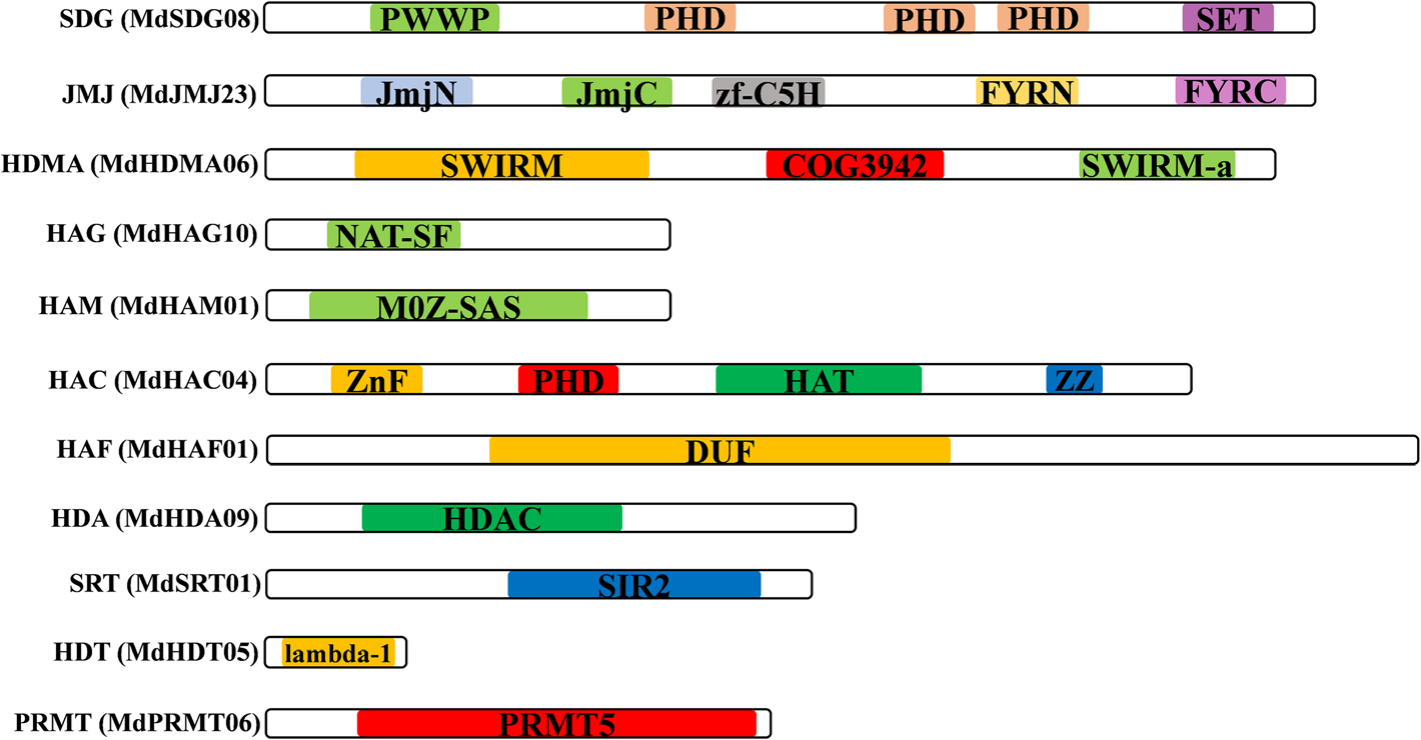
Diagrammatic shows of the representatives of each *MdHMs* gene family

As mentioned above and (Additional file 7: Figure S2) shown, different HMs genes had different typical domains. We then investigated the structures of the 11 kinds of *HMs* gene families to confirm the present of each domain in apple, and a random gene was selected and to analyze their DOMAIN structure (Fig. 6). The HMs proteins shared various structures, with MdSDG08 containing a PWWP, PHD, and SET, MdJMJs containing a JmjN, JmjC, zf-C5H FYRN, and FYRC, MdHDMAs containing a SWIRM, COG3942, and SWIRM-a, MdHAGs containing a NAT-SF, MdHAMs containing a M0Z-SAS, MdHACs containing a ZnF, PHD, HAT, and ZZ, MdHAF containing a DUF, MdHDA containing a HDAC, MdSRT containing a SIR2, MdHDT containing a lambda-1, and MdPRMTs containing a PRMT5. These identified structures were similar to those found previously in the *HMs* of *Citrus* and other plants, indicating their conserved evolution.

Gene structures and motifs also play important roles during gene evolution. Therefore, we performed detailed exon-intron structure and protein motif analysis for t seven candidate gene families. Seven individual phylogenetic trees (MdSDGs, MdPRMTs, MdHDMAs, MdJMJs, MdHAGs, MdHDAs and MdHDTs) were built based on protein sequences (Additional file 8: Figure S3, Additional file 9: Figure S4, Additional file 10: Figure S5, Additional file 11: Figure S6, Additional file 12: Figure S7, Additional file 13: Figure S8, Additional file 14: Figure S9 and Additional file 15: Table S6). As shown in Additional file 8: Figure S3, the proteins encoded by the *SDGs* gene family (*MdSDG43*, *57*, *51*, *50*, *49*, and *28*), which shared similar structures, were closely clustered. Additionally, 13 MdSDGs family members (MdSDG25, 26, 29, 59, 45, 35, 19, 24, 14, 39, 47, 03, 61, 29, 43, and 57) shared the greatest number of motifs compared with the other MdSDGs proteins. In addition, MdPRMT05, 02, 07 and 01 also shared similar protein motifs (Additional file 9: Figure S4). MdHDMAs also showed conserved motifs. For example, MdHDMA03, 12, 07, 08, 13, 06, 11, 02, and 01 shared only motif 1, 6, and 7; while the remaining MdHDMAs proteins, except MdHDMA09, shared more motif. MdHDMA14 and MdHDMA9 were encoded by genes having similar structures (Additional file 10: Figure S5). Gene structures and protein motifs of the MdJMJs were similar to MdHDMAs. For example, 9 MdJMJs proteins (MdJMJ17, 08, 03, 22, 06, 10, 14, 09, and 20) shared similar motifs. In addition, their gene structures showed less variability, especially among closely connected genes (*MdJMJ9* and *MdJMJ20*, *MdJMJ27* and *MdJMJ16*, *MdJMJ23* and *MdJMJ7*, and *MdJMJ28* and *MdJMJ11*) (Additional file 11: Figure S6, Additional file 15: Table S6).

The structures of one *HATs* and two *HDACs* gene families were also determined. Among the *MdHAGs* gene family members, most of them shared only one CDS (Additional file 12: Figure S7). For example, *MdHAG18*, *19*, *17*, *08*, *14*, *34*, *49*, *38*, *13*, *33*, and *01* contained a coherent CDS within their gene structures. Their motifs were also conserved among some closely related genes, as seen with other *HM*s. As for *HDACs* and *HDTs*, they also had similar gene structures and encoded proteins with similar motifs, such as *MdHDA15* and *MdHDA09*, *MdHDA10* and *MdHDA16*, *MdHDT01* and *MdHDT03*, and *MdHDT02* and *MdHDT04* (Additional files 13-14: Figure S8 and S9).

### Analysis of *HMs* orthologous genes against in other species

**Fig. 7:**
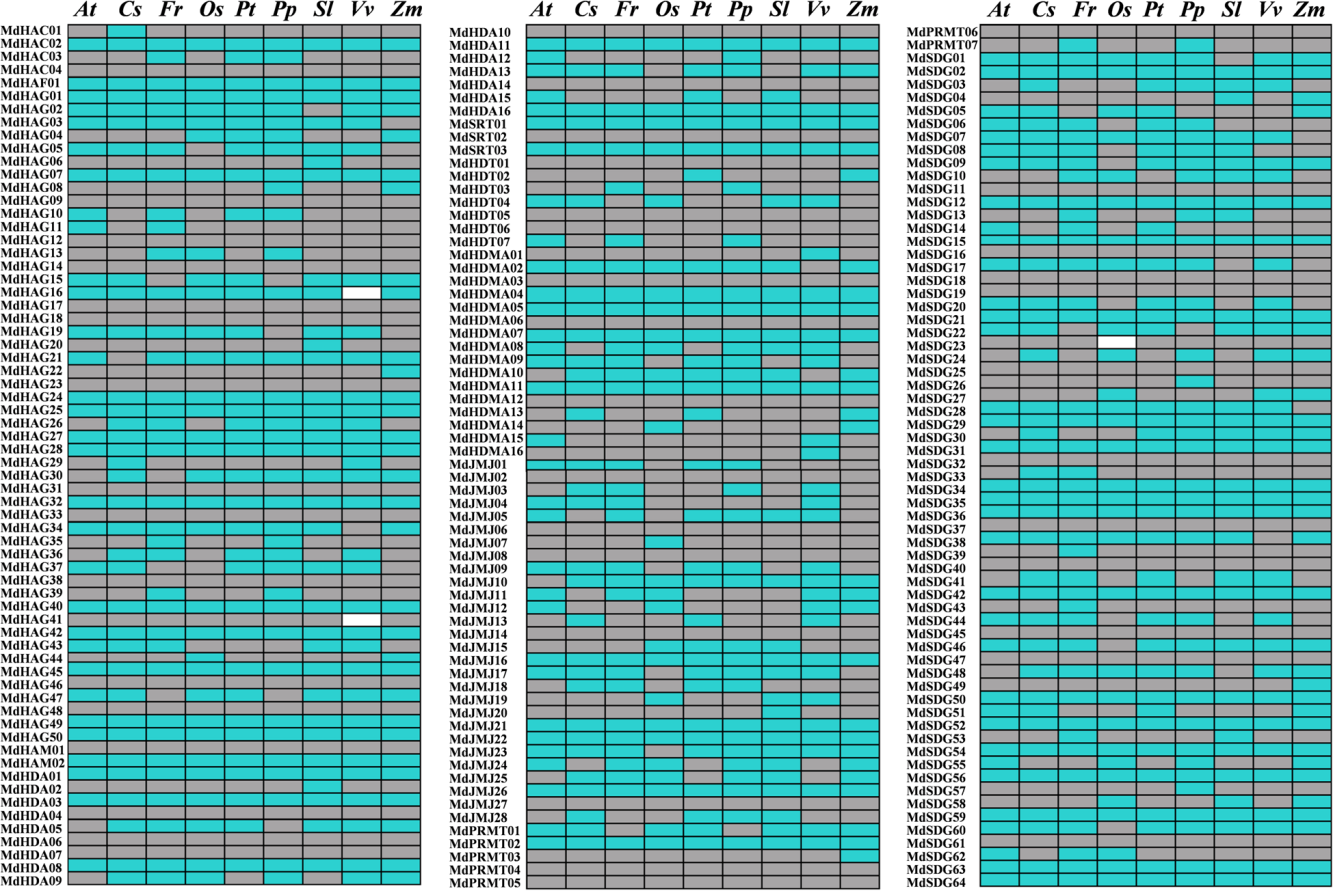
Apple *HMs* genes orthology against with sequenced species. Blue, a one to one ortholog *HM* in the candidate species; Gray, *MdHMs* has orthology in the candidate species but it was not one to one detected; White, no detected. *At*, *Arabidopsis thaliana*; *Cs*, *Citrus sinensis*; *Fv*, *Fragaria vesca*; *Os*, *Oryza sativa*; *Pt*, *Populus trichocarpa*; *Pp*, *Prunus persica*; *Sl*, *Solanum lycopersicum*; *Vv*, *Vitis vinfera*; *Zm*, *Zea mays*

BLASTP algorithm was employed to identify *MdHM*s orthologous genes with other sequenced plant species, and they were identified with e-value lower than 1e-20 and sequence homology more than 60%, as previous reported methods [44]. The 10 candidate plants used were including *Arabidopsis*, *Zea may*, *Solanum lycopersicum*, *Oryza sativa*, *Citrus*, *Vitis vinifera* and *Populus trichocarpa*, as well as the Rosaceae plants *Fragaria vesca*, *Prunus persica*, and *Pyrus sorotina.* Their orthologous relationships were divided into three kinds [44]: a) genes that existed in apple and were absent from a given species; b) apple genes that had one to one orthologs in a given species; and c) apple genes that had homologs in a given species but not orthologs. As shown in Fig. 7, most *MdHM*s had homologous genes compared with the 10 candidate species. However, *MdHAG16* and *MdHAG24* had no homolog in *V. vinifera*, nor *MdSDG23* in *O. sativa*.

### Interactions prediction of MdHMs protein

To further predict their biological interactions, we visualized HM proteins with Cytoscape v3.5.1 [45, 46]. As shown in Additional file 16: Figure S10, Additional file 17: Table S7 members from four HM-related clusters, − HMTs, HDMs, HATs, and HDACs, directly or indirectly interacted with other proteins. Among them, the HMTs interacted with the greatest number of proteins (25), followed by the HDACs (11). The HDMs and HDACs only interacted with five and four proteins, respectively. Some proteins, such as MdSDG29, MdHAM02, MdJMJ01, MdJMJ25, MdHAG28, and MdSDG14, could also directly or indirectly interact with at least three kinds of proteins. Totally, *MdHMs* regulated downstream genes or were regulated by their up-regulated genes to participate in various processes.

### Expression profiles of *MdHMs* with high-throughput sequencing

**Fig. 8:**
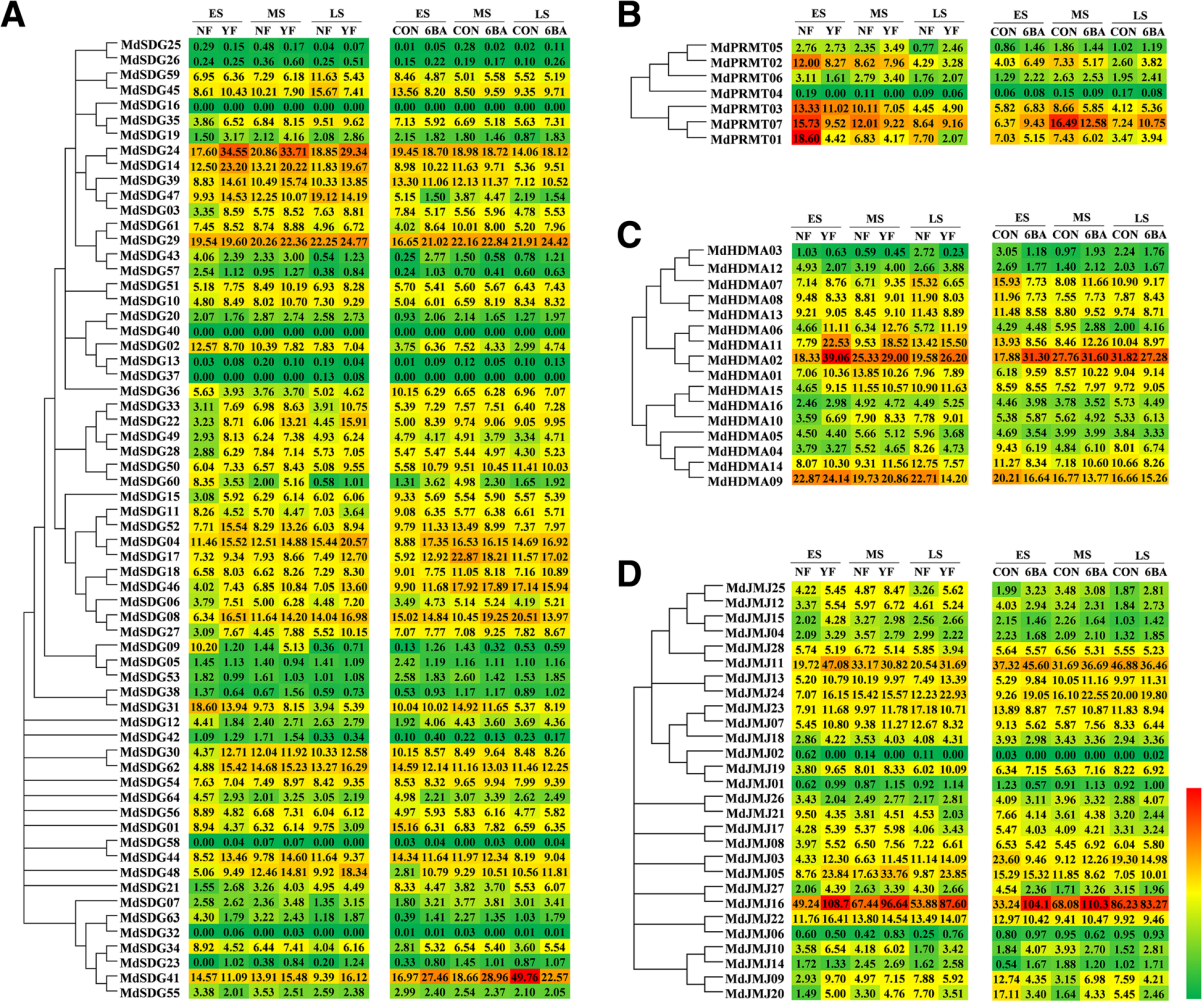
Expression profiles of *MdHMTs* and *MdHDMs* during flower induction period in two different varieties and 6BA treatment. **a** *MdSDGs* gene families; **b** *MdPRMTs* gene families; **c** *MdHDMAs* gene familes; **d** *MdJMJs* gene families. FPKM values were used to generate their expression profiles

**Fig. 9:**
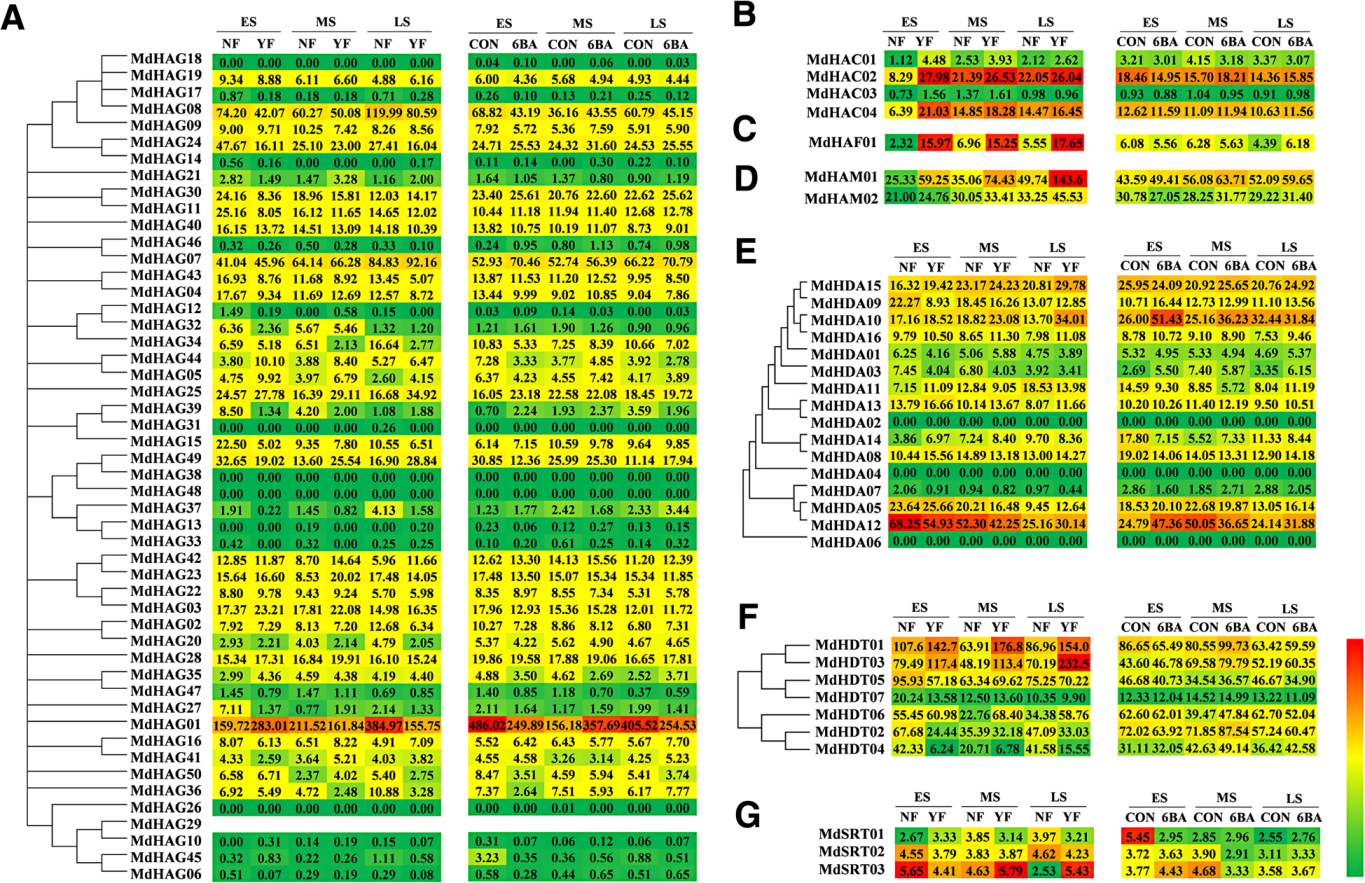
Expression profiles of *MdHATs* and *MdHDACs* during flower induction period in two different varieties and 6BA treatment. **a** *MdHAGs* gene families; **b** *MdHACs* gene families; **c** *MdHAFs* gene families; **d** *MdHAMs* gene families; **e** *MdHDAs* gene families. FPKM values were used to generate their expression profiles. **f** *MdHDTs* gene families, **g** *MdSRTs* gene families

To better understand their potential involvement in responses to flower induction, we used published transcriptome data to evaluate the 198 *MdHMs* expression profiles [31]. FPKM (Fragments Per Kilobase of transcript sequence per Millions mapped reads) was calculated to assess gene expression levels. The resulting *p* value were then adjusted with Benjamini and Hochberg’s approach for controlling the false discovery rate, and a corrected *P* value of 0.05 and log2 value (fold change) of 1 were set as the criteria for identifying DEGs. [31]. Treatments with 6-BA increase the ratios of short shoots and result in higher flowering rates. Additionally, ‘Yanfu No.6’ has a higher flowering rate than ‘Nagafu No.2’ [31, 35]. We analyzed the candidate *MdHMs* expression levels in response to exogenous 6-BA treatments and in two varieties with different flowering capabilities, Nagafu No.2 and Yanfu No.6. As shown in Figs. 8 and 9, respectively. Of the 198 *MdHM*s, 197 genes, with the exception of *MdHAG29*, were detected in our transcriptome sequencing (Figs. 8 and 9). Of the 197 detected genes, 28 genes, 7 *MdSDG*s (*MdSDG26*, *16*, *40*, *13*, *37*, *58*, and *32*), 1 *MdPRMT* (*MdPRMT4*), 3 *MdJMJ*s (*MdJMJ02*, *01*, and *06*), 13 *MdHAG*s (*MdHAG18*, *17*, *14*, *46*, *31*, *38*, *48*, *13*, *33*, *26*, *10*, *45*, and *06*), 1 *MdHAC* (*MdHAC03*), and 3 *MdHDA*s (*MdHDA02*, *04*, and *06*) showed no or very low expression levels (less than 1). The non-existent or low expression levels of these *MdHM*s indicated that they did not function to great degrees in flower development. On the contrary, the expression levels of six genes, *MdJMJ16*, *MdHAG08*, *MdHAG01*, *MdHAM01*, *MdHDT01*, and *MdHDT03*, were extremely high (greater than 100), indicating that they may have important roles during the flower-induction period (Figs. 8 and 9).

The different expression patterns of the varieties Nagafu No.2 and Yanfu No.6 were also analyzed. In the easy-flowering variety Yanfu No.6, 27 *MdSDGs* genes, *MdSDG07*, *29*, *27*, *48*, *35*, *19*, *24*, *14*, *39*, *03*, *61*, *29*, *51*, *10*, *33*, *22*, *49*, *50*, *52*, *04*, *17*, *18*, *46*, *06*, *08*, *27*, and *62*, were highly expressed. However, only three *MdSDG* genes *MdSDG55*, 59, and *36*, and two *MdPRMT* genes (*MdPRMT02* and *MdPRMT02*) were more highly expressed in variety Nagafu No.2 (Fig. 8). Additionally, 12 *MdHDM*s, *MdHDMA06*, *11*, and *02*, and *MdJMJ25*, *12*, *24*, *19*, *03*, *05*, *16*, *22*, and *10*, were higher in ‘Yanfu No.6’ while three *MdHDM*s, *MdHDMA05*, *MdHDMA04*, and *MdJMJ28*, were higher in ‘Nagafu No.2’.

The different expression patterns of the varieties Nagafu No.2 and Yanfu No.6 were also analyzed. In the easy-flowering variety Yanfu No.6, 27 *MdSDG* genes, *MdSDG07*, *29*, *27*, *48*, *35*, *19*, *24*, *14*, *39*, *03*, *61*, *29*, *51*, *10*, *33*, *22*, *49*, *50*, *52*, *04*, *17*, *18*, *46*, *06*, *08*, *27*, and *62*, were highly expressed. However, only three *MdSDG* genes *MdSDG55*, 59, and *36*, and two *MdPRMT* genes (*MdPRMT02* and *MdPRMT01*) were more highly expressed in variety Nagafu No.2 (Fig. 8). Additionally, 12 *MdHDM*s, *MdHDMA06*, *MdHDMA11*, *MdHDMA02*, *MdJMJ25*, *12*, *24*, *19*, *03*, *05*, *16*, *22*, and *10*, were higher in ‘Yanfu No.6’, while three *MdHDM*s, *MdHDMA05*, *MdHDMA04*, and *MdJMJ28*, were higher in ‘Nagafu No.2’ (Figs. 8 and 9).

### qRT-PCR analysis of candidate *MdHMs* genes

**Fig. 10:**
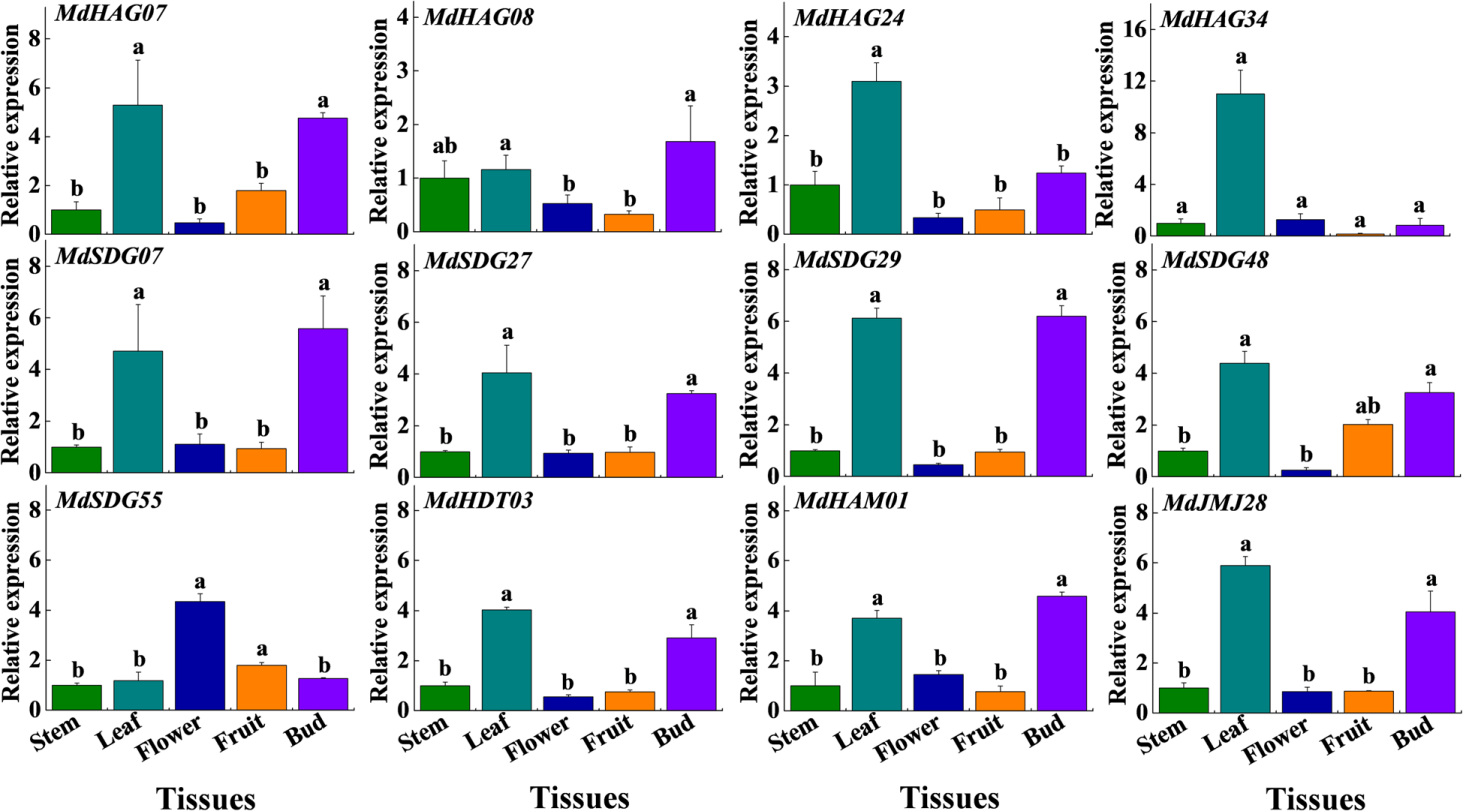
Transcript levels of 12 *MdHMs* genes among different tissues by qRT-PCR. Tissues were collected from Nagafu No.2. Each value represents the mean ± standard error of three replicates. Means followed by small letters are significantly different at the 0.05 level (the same below)

In total, 12 *MdHM*s (*MdHAG07*, *08*, *24*, and *34*, and *MdSDG07*, *27*, *29*, *48*, and *55*, *MdHDT03*, *MdHAM01*, and *MdJMJ28*) were selected and their expression levels assessed using qRT-PCR under different flowering-related circumstances and in different tissues (stems, leaves, flowers, fruits, and buds). These candidate *MdHM*s showed different expression patterns in the five tissues, and 11 of them showed higher levels in leaves and buds, with the exception of *MdSDG55*, which was higher in flowers (Fig. 10). The 12 candidate *MdHM*s’ responses to various hormones (GA3, ABA, SA, and MeJA) were also investigated (Additional file 18: Figure S11). *MdHAG34* and *MdSDG55* were not sensitive to these hormone treatments. The remaining 10 genes were up- or down-regulated at different time points after treatment, indicating that they might have roles in hormone stress responses or apple development.

**Fig. 11:**
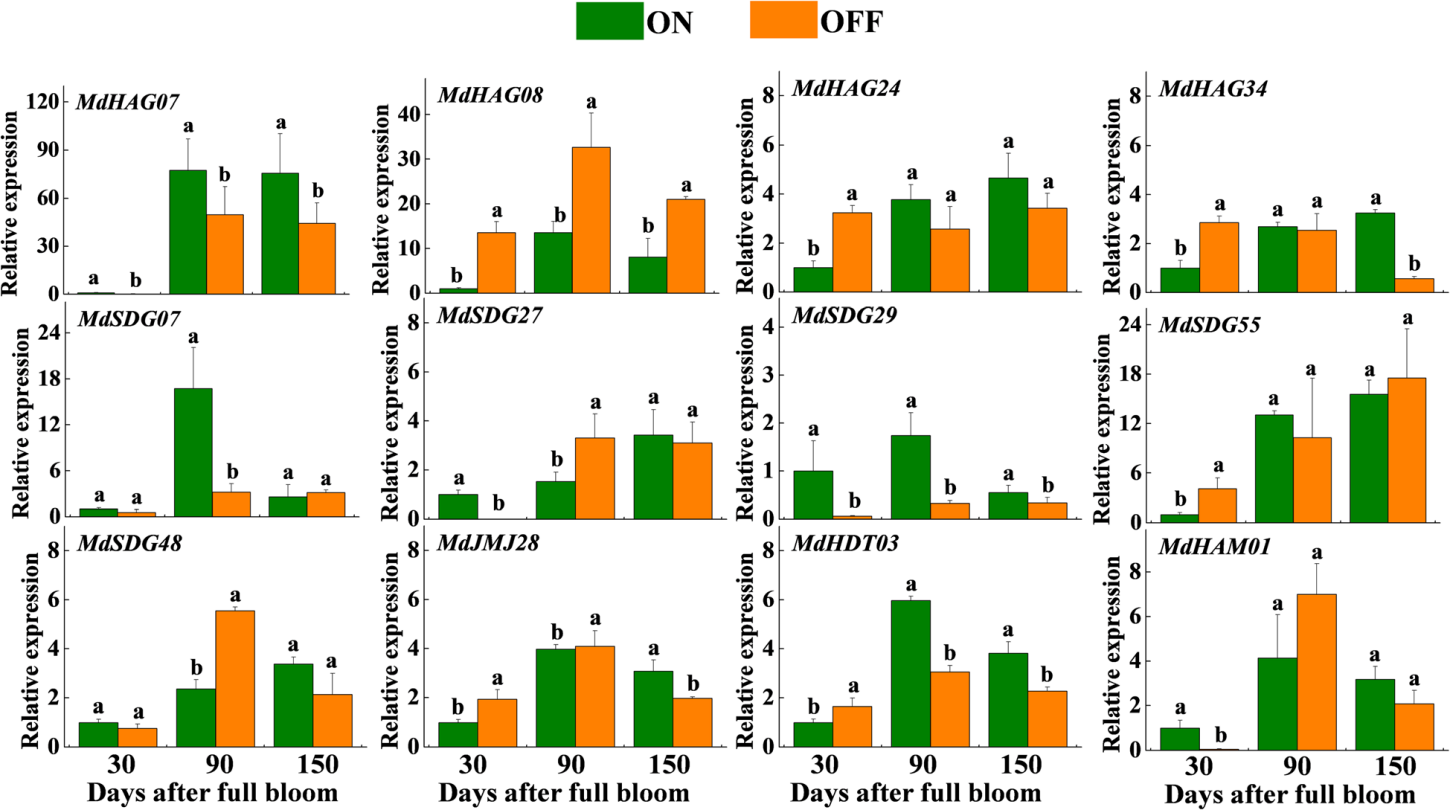
Transcript levels of 12 *MdHMs* genes in alternate bearing trees by qRT-PCR. Terminal buds were collected from 30, 90 and 150 DAFB

Exogenous sugar treatments can promote flowering and lead to a higher flowering rate [32]. Here, we analyzed the expression profiles of the genes after sugar treatments. As seen in Additional file 19: Figure S12, these candidate genes were also responsive to sugar-mediated flowering induction during the flower-induction period, especially at 70 DAFB. For example, most candidate genes, such as *MdHAG07*, *MdHAG08*, *MdSDG29*, *MdSDG55*, *MdSDG48*, *MdHDT03*, and *MdHAM01*, showed different expression patterns at 70 DAFB after the sugar treatment. We further analyzed their expression profiles under different flowering-related circumstances (i.e., sugar treatments and alternate bearing). We investigated their expression levels in alternate-bearing ‘Fuji’ trees. The 12 *MdHM*s were expressed during the flowering periods of both the ‘ON’ and ‘OFF’ tree buds (Fig. 11). Among them, the *MdHAG08* level was higher in the ‘OFF’ year in all three developmental stages, while those of *MdHAG07* and *MdSDG29* showed the opposite trend. *MdHAG24*, *MdHAG34*, and *MdHDT03* were expressed higher at 30 DAFB in ‘ON’ trees but decreased at 90 and 150 DAFB. The expression patterns of *MdSDG07* and *MdJMJ28* also differed. At 30 and 90 DAFB, *MdSDG07* was higher in ‘ON’ trees and then decreased, while *MdJMJ28* was higher in ‘OFF’ trees and then decreased. Thus, the 12 *MdHM*s appeared to be involved in sugar- or hormone-mediated flower induction, as well as in alternate bearing.

## Discussion

*HMs* played important roles during plant growth and development processes. Although, great advances have been made in some model plants, less has been reported in fruit trees except water deficit and fruit development traits [38, 47, 48]. Here, 198 *MdHMs* genes, 71 *MdHMT*s (64 *MdSDG*s and 7 *MdPRMT*s), 44 *MdHDM*s (16 *MdHDMA*s and 28 *MdJMJ*s), 57 *HAT*s (50 *MdHAG*s, 2 *MdHAM*s, 4 *MdHAC*s, and 1 *MdHAF*), and 26 *MdHDAC*s (16 *MdHDA*s, 3 *MdSRT*s, and 7 *MdHDT*s), were identified in the apple genome. They were further characterized, including gene phylogeny, protein-protein interactions, and expansion and synteny analyses. In addition, we investigated their potential expression levels and roles in response to flower induction. These results will add to the knowledge in this field.

### Comparison *HMs* genes in apple and other sequenced plant species

Identification of *HMs* genes, including *HMT*s (*SDG*s and *PRMT*s), *HDM*s (*HDMA*s and *JMJ*s), *HAT*s (*HAG*s, *HAM*s, *HAC*s, and *HAF*s), and *HDAC*s (*HDA*s, *SRT*s, and *HDT*s) have been systematically or partially reported in *Citrus*, *S. lycopersicum*, *Arabidopsis*, *Z. mays*, and *O. sativa* [3, 6, 7, 49]. Little is known about *HMs* gene families in the important economic apple trees. With the republication of apple genome, it is useful for us to explore more information for genomic analysis. In present study, we totally identified 198 putative *MdHMs* (Table 2). They were divided into four classifications (*HMT*, *HDM*, *HAT*, and *HDAC*), and they belonged to 11 different subfamilies, which were similar to those of other species [6, 7]. Of all the *HMs*, *SDGs* were the most conserved among the species. The number of *MdSDGs* was nearly 1.5-fold greater than the numbers of *SlSDG*s, *CsSDG*s, *AtSDG*s, *OsSDG*s, and *ZmSDG*s. Additionally, the number of *MdHDMAs* genes was nearly two to four times greater than those of other species. The number of *HAG*s in apple was greatly different from other species, especially *Arabidopsis*, *O. sativa* and *Z. mays.* This great difference was partially diminished when the AT1 domain was used as the query in a BLAST algorithm-based search, which led to 33 *HAG*s being identified in *Arabidopsis* [4]. Other genes, such as *JMJ*s, *HAD*s, and *HDT*s, were also present two times more in apple than in other species (Table 2). We also searched orthologous genes against in other species of *HMs* genes (Fig. 7), which would be a useful tool for further analysis.

**Table 2:** Summary of *HMs* gene families in different plants

Types	*Malus domestica*	*Solanum lycopersicum*	*Citrus sinensis*	*Aradopsis thaliana*	*Oryza sativa*	*Zea mays*
*HMTs*						
*SDG*	64	43	40	41	37	38
*PRMT*	7	9	7	7	5	5
*HDMs*						
*HDMA*	16	6	3	4	4	4
*JMJ*	28	20	20	20	20	10
*HATs*						
*HAG*	50	26	45	3	3	4
*HAM*	2	1	1	2	1	2
*HAC*	4	4	2	5	3	5
*HAF*	1	1	2	2	1	1
*HDACs*						
*HDA*	16	9	9	12	14	11
*SRT*	3	2	5	2	2	1
*HDT*	7	3	2	4	2	4

The density of apple *HM*s was followed by *Arabidopsis* and citrus (Additional file 20: Table S8). *Arabidopsis* were the most redundant, while *Z. mays* were the least dense, which might be the result of its large genome size [50]. The apple genome is nearly 1.7 times larger than that of citrus, but their gene numbers are not positively correlated with genome size. Similar relationships exist in other plants (Table 2). The complex connections between genome size and gene number are not well characterized, partially because of duplication events in the genomes of different species and/or their complicated species characterizations. Meanshile, 198 *MdHM*s were not equally distributed on the 18 chromosomes of the apple genome (Fig. 1), similar to those of citrus [6]. Additionally, this irregular distribution was noticed for the *MdGRAS* and *MdGASA* gene families [33, 35]. Theoretically, apple chromosome 15 was longer and larger than other chromosomes, it could easily contain more genes (Fig. 2) [38].

The gain or loss of an exon or intron is always associated with the diversification of gene families. These events can be caused by chromosomal rearrangements or fusions, and they can result in distinct functional characterizations [51]. In the present study, *MdHMs* genes with different structures were always distantly clustered, while genes with similar structures were tightly clustered (Additional file 8: Figure S3, Additional file 9: Figure S4, Additional file 10: Figure S5, Additional file 11: Figure S6, Additional file 12: Figure S7, Additional file 13: Figure S8 and Additional file 14: Figure S9). This was also observed in the *IDD* and *GASA* gene families in the apple genome, indicating potential relationships among phylogeny, gene structures, and protein motifs [33–35]. The typical domains of gene clusters in 12 *MdHM*s were investigated. Generally, these domains were conserved in apple (Fig. 6). For example, the SET domain is conserved in other plants [6, 7, 52], and in apple, MdSDGs also shared this typical SET domain. Additionally, other dispensable domains were also found in MdSDG family members, as in citrus, which shared 19 different domains among its 40 CsSDGs [6]. All of the MdPRMT shared a typical PRMT5 domain. When compared with citrus and tomato, JmjC and SWIRM were also typical conserved domains in the JMJ and HDMA gene families, respectively (Fig. 6) [6, 7]. Similar typical structures are found in other family members among different species. For example, the AT-N domain is in the HAGs, C-terminal MOZ-SAS is in the HAMs, HD is in the HDAs, and SIR2 is in the SRTs (Fig. 6, Additional file 7: Figure S2). Although their sequence characterizations and structures were varied and numerous, their prerequisite domains were conserved, indicating a common characteristic that was conserved among various species [4, 6, 7].

### Evolution and expansion analysis of *HM* gene family

To better understand their phylogenetic interactions, four phylogenetic trees (*HMT*s, *HDM*s, *HAT*s, and *HDAC*s) were constructed using the all of the gene members from apple and the model plant *Arabidopsis*. Interestingly, one *PRMT* gene, *AtPRMT16* was clustered with other *SDG* genes (Fig. 3a), which might be caused by their partly matching protein sequences. The *HDM*s, *HAT*s, and *HDAC*s were also clustered. Among the *HDM*s, the subfamily *HDMA* clustered separately with the *JMJ* subfamily (Fig. 3b). The remaining *HAT*s and *HDAC*s were well organized and clustered in a logical fashion, as previous found in other species [6, 7]. Totally, we firstly analyzed the subfamilies within the four trees, *HMT*s (*SDG*s and *PRMT*s), *HDM*s (*HDMA*s and *JMJ*s), *HAT*s (*HAG*s, *HAM*s, *HAC*s, and *HAF*s), and *HDAC*s (*HDA*s, *SRT*s, and *HDT*s). The emergence of four different trees from *HM* subfamilies was useful to investigate their complex phylogenetic interactions.

Gene duplication contributed to the evolution of species [51]. Additionally, in apple, a recent (more than 50 million years ago) whole-genome duplication event took place, which led to a change of apple chromosomes from 9 (ancestral) to 17 (present) [53]. Using improved sequencing technology, a new version of the apple genome was recently published [38]. In the present reference genome, many identified *MdHMs* showed duplicated genes according to the Circos diagram (Fig. 4). In tomato, eight *SlHAGs* gene members, including *SlHAG11*, *19*, *20*, *21*, *22*, *24*, *25*, and 26, underwent tandem duplications [7]. Additionally, *SlHAC*s, *SlSDG*s, and other subfamilies were also analyzed for gene duplications, as in our study. In apple, it was reported that *MdSPL*, *MdGASA*, and *MdGRAS* also experienced tandem, segmental duplications or whole genome duplications, similar as the *MdHMs* family members [33, 35, 36]. This gene duplications or gene expansion was associated with the genome duplication [53].

Previously, the Ka/Ks ratio was determined to be a good indicator of positive selection (Ka/Ks > 1), neutral selection (Ka/Ks = 1), or purifying selection (Ka/Ks < 1) [54, 55]. Interestingly, our duplicated gene pairs within apple, or between apple and *Arabidopsis*, were all less than 1 (Fig. 7), which was similar to a previous report for *WRKY* in *Brachypodium distachyon* [46], indicating their important relationships during evolution. Totally, these duplications were associated with the expansion of *MdHM*s, led to their diverse structures and functions.

The synteny between duplicated blocks in *Arabidopsis* and apple was also determined of the *HM*s. Because *Arabidopsis* is a model plant and functions of the *AtHM*s are better understood. A comparative genomic comparison investigation helped us understand information on *AtHM*s to *MdHM*s, and possible functions of *MdHMs* can be well inferred [56, 57]. Here, several orthologous genes were also detected in the syntenic maps, and these orthologous genes were located in different duplicated genomic regions of the *Arabidopsis* and apple genome (Fig. 6), indicating that these genes were derived from a common ancestor. Previously, *AtHMs* genes, including, *AtSDG8* [9], *AtHDA9* [8], *AtHDA19* [17], *AtHDT1* [58], *AtHDT3* [59], *AtHDA15* [60], *AtHAM1* and *2* [19, 20], *AtHAF1* [61], and *AtSRT1* and *2* [62, 63], were shown to be involved in flower induction. Therefore, based on the orthologous genes between apple and *Arabidopsis*, several *MdHM*s could be inferred according to their *Arabidopsis* comprasion. However, these need to be confirmed by further experiments.

### *MdHMs* were putatively involved in apple flower induction

Histone modifications related genes in plants have been reviewed [12, 13]. Like transcription factors, the *HMs* genes were also involved in various biological processes during plant growth and development, especially flower induction [64–66]. Various genes and gene families involved in flowering have been well characterized in plants. In apple, the *MdMAD-box*, *MdIDD*, *MdGASA*, and *MdGRAS* gene families were involved in regulating apple flowering [33–35, 37]. However, whether *MdHM*s respond to flower induction was reported. Here, we proposed that *MdHM*s were also responsible for flower induction in apple. In the model *Arabidopsis*, several *HM* genes, such as *AtSDG8* [9], *AtHDA9* [8], *AtHDA19* [17], *AtHDT1* [58], *AtHDT3* [59], *AtHDA15* [60], *AtHAM1* and *2* [19, 20], *AtHAF1* [61], and *AtSRT1* and *2* [63, 64] have been functionally confirmed, and they are involved in flower development. Thus, we identified candidate apple flowering-related genes by referring to their orthologous genes and their expression patterns. For example, *MdHDA13*, orthologous to *AtHDA9*, showed a consistent expression pattern during the flower stages and was expressed higher under higher flowering circumstances (‘Yanfu No.6’ and 6BA treatment). Similarly, *MdHDA16*, an orthologs of *AtHDT15*; *MdHAM01*, an orthologs of *AtHAM1*; and *MdHAF01*, an orthologs of *AtHAF1*, were expressed higher in ‘Yanfu No.6’ than in ‘Nagafu No.2’. However, *MdHDT04*, an orthologs of *AtHDT1*, was more highly expressed in ‘Nagafu No.2’ (Figs. 8 and 9). Thus, this comparative analysis of *HMs* genes in apple and *Arabidopsis*, together with their expression patterns, provided valuable information for the involvement of *MdHM*s in regulating flower induction.

Leaves and buds are important organs that influence flower development [28, 29, 67]. Here, 11 of the candidate *MdHMs* were expressed higher in leaves or buds than in other tested tissues (stems, flowers and fruits), which indicated their involvement in flowering. We analyzed their expression patterns in two varieties Nagafu No.2 and Yanfu No.6. ‘Yanfu No.6’ is a ‘Nagafu No.2’ mutant that has a higher proportion of spurs, shorter shoots, larger buds and a higher flowering rate [34, 35]. Most of the *MdHM*s were expressed and showed consistent patterns during the three developmental stages. A majority of the *MdSDG* genes were higher in ‘Yanfu No.6’ during the flower development stages (Fig. 8), indicating that methylation is occurring in ‘Yanfu No.6’ and ‘Nagafu No.2’. Similarly, higher acetylation-related activities occurred in ‘Nagafu No.2’ (Fig. 9). Similar epigenetic interactions were also reported among some somatic mutations [68–70]. Therefore, we speculated that the up- or down-regulation of *MdHM*s contributed to different flowering phenomena, which directly or indirectly affected flowering. The continuous differential expression patterns of *MdHM*s could partly illustrate their modification processes and affect flowering. We also determined their expression levels in response to sugar treatments and hormonal stresses (Additional files 18-19: Figure S11 and S12). In general, they were also partly involved in sugar-mediated flower induction in apple.

Although crosstalk about hormones or sugar-mediated alternate bearing has been reported in perennial trees, unsloved problems were still remained [29, 71, 72]. Here, we investigated the expressions of 12 candidate *MdHM*s in alternate bearing apple trees. With less reported literature about *HMs* and alternate bearing, we could not make better propose about this. But they were indeed induced and showed different expression patterns in ‘ON’ and ‘OFF’ trees at different time points, indicating that they were responsible for different development stages (Fig. 11). Further researches needed to be performed to confirm this.

## Conclusions

In this study, we systematically identified *HMs* genes in the apple genome. Their chromosome locations, gene and protein structures, phylogenetic and synteny relationships, and protein-protein interactions were also characterized. Their expression levels in different flowering ability varieties and 6BA treatment were also investigated using high-throughput RNA sequence data in the apple buds, indicated they were responsible to flower induction. Further some candidate *HMs* genes were then analyzed by qRT-PCR in different tissues (stems, leaves, flowers, fruits, and buds), in different hormones stresses (GA3, ABA, SA and MeJA), and different flowering related circumstances (sugar treatment and alternate bearing buds). Totally, our identification and characterization of *HMs* genes in apple provided useful information and enriched biological theories, which could be foundation for further analysis.

## Additional files

Additional file 1: **Table S1.** List of Pfam accession number of each *HMs* gene family (DOCX 13 kb)

Additional file 2: **Table S2.** Primer information for gene expression analysis (DOCX 13 kb)

Additional file 3: **Table S3.** Table S3. Detailed annotations of *MdHMs* according to the Gene Ontology (GO) terms (XLS 354 kb)

Additional file 4: **Table S4.** Synteny analysis of *MdHMs* genes (DOCX 26 kb)

Additional file 5: **Table S5.** Synteny information of *MdHMs* and *AtHMs* genes (DOCX 25 kb)

Additional file 6: **Figure S1.** Average Ka, Ks values of duplication gene pairs. (A) gene pairs of apple; (B) gene pairs of apple and *Arabidopsis* (TIF 367 kb)

Additional file 7: **Figure S2.** Diagram of HMs typical conserved domains (TIF 846 kb)

Additional file 8: **Figure S3.** Gene structure and protein motifs analysis of *MdSDGs* gene family members (TIF 3270 kb)

Additional file 9: **Figure S4.** Gene structure and protein motifs analysis of *MdPRMTs* gene family members (TIF 571 kb)

Additional file 10: **Figure S5.** Gene structure and protein motifs analysis of *MdHDMAs* gene family members (TIF 639 kb)

Additional file 11: **Figure S6.** Gene structure and protein motifs analysis of *MdJMJs* gene family members (TIF 1445 kb)

Additional file 12: **Figure S7.** Gene structure and protein motifs analysis of *MdHAGs* gene family members (TIF 1174 kb)

Additional file 13: **Figure S8.** Gene structure and protein motifs analysis of *MdHDAs* gene family members (TIF 1253 kb)

Additional file 14: **Figure S9.** Gene structure and protein motifs analysis of *MdHDTs* gene family members (TIF 429 kb)

Additional file 15: **Table S6.** Motif sequences of *MdHMs* proteins (DOCX 16 kb)

Additional file 16: **Figure S10.** Interaction networks analysis of *MdHMs* genes (TIF 1052 kb)

Additional file 17: **Table S7.** Predication protein-protein interaction information according to their orthologous in *Arabidopsis* (XLS 59 kb)

Additional file 18: **Figure S11.** Transcript levels of 12 *MdHMs* genes following GA3, ABA, SA, and MeJA in by qRT-PCR. Leaves were collected after 0, 3,6 and 12 h after treatment. Each value represents the mean ± standard error of three replicates. Means followed by small letters are significantly different at the 0.05 level (the same below). (TIF 3532 kb)

Additional file 19: **Figure S12.** Transcript levels of 12 *MdHMs* genes following sugar treatment and in Yanfu No.6. Terminal buds were collected from 30, 50, and 70 DAFB. (TIF 734 kb)

Additional file 20: **Table S8.** Summary of *HMs* in different species (DOCX 13 kb)

## Abbreviation

6BA: 6-benzylaminopurine
ABA: Abscisic acid
DAFB: Days after full bloom
HAT: Hours after treatment
HATs: Histone acetylases
HDACs: Histone deacetylases
HDMs: Histone methylases
HM: Histone modification
HMTs: Histone methyltransferases
MeJA: Methyl jasmonate
SA: Salicylic acid
