# Supplementary material for: Identification and characterization of histone modification gene family reveal their critical responses to flower induction in apple
Source: BMC Plant Biol. 2018 Aug 20;18:173. doi: 10.1186/s12870-018-1388-0 (PMC6102887; doi:10.1186/s12870-018-1388-0)
Supplement: Supplementary file 1 — Table S1. List of Pfam accession number of each HMs gene family (DOCX 13 kb) [file 12870_2018_1388_MOESM1_ESM.docx]

Table S1. List of Pfam accession number of each *HM* gene family.

| Gene type | Family name | Accession number |
| --- | --- | --- |
| *HMTs* | *SDGs* | PF00856 |
|  | *PRMTs* | PF05185 |
| *HDMs* | *HDMAs* | PF04433 |
|  | *JMJs* | PF02373 |
| *HATs* | *HAGs* | PF00583 |
|  | *HAMs* | PF01853 |
|  | *HACs* | PF08214 |
|  | *HAFs* | PF09247 |
| *HDACs* | *HDAs* | PF00850 |
|  | *SRTs* | PF02146 |
|  | *HDTs* | / |
