# Supplementary material for: Identification and characterization of histone modification gene family reveal their critical responses to flower induction in apple
Source: BMC Plant Biol. 2018 Aug 20;18:173. doi: 10.1186/s12870-018-1388-0 (PMC6102887; doi:10.1186/s12870-018-1388-0)
Supplement: Supplementary file 2 — Table S2. Primer information for gene expression analysis (DOCX 13 kb) [file 12870_2018_1388_MOESM2_ESM.docx]

Table S2. Primer information for gene expression analysis.

| Gene name | Forward primer (5’-3’) | Reverse primer (5’-3’) |
| --- | --- | --- |
| *MdHAG08* | GATGGAGGAGGAGAGCAACGAGTG | GTACAAATTGAACGCCGCCCGATT |
| *MdHAG07* | CGCCAAGCCGCTCTTCTCTTAC | TCGCAATCGCTCTCGCTATCCT |
| *MdHAG34* | CTTCAACCTCCACCGCACGATC | AACCTCTCCATCACCGCCTTCC |
| *MdHAG24* | CGGCGGCGTTGGAGATAGAGTA | AACAGAGCTGCGGCCACAGA |
| *MdSDG07* | ATGGAGTTGGCGATGCTGCAAA | GCGAGGAAGCCAGCGAATGTA |
| *MdSDG29* | TGCTGCGGACAACATACCTCCT | GTGGCACCACAATGGCAGAACA |
| *MdSDG27* | GCAACGGCGTGGAAGGCATT | ACCTGTGTGTCGCTCCCATTCA |
| *MdSDG55* | CTTTACCGCCTTCCGCCCTCTA | GCGAGCCAAATCGTCGAGAACA |
| *MdSDG48* | CGCTGCGAAGTCACTCAGTTGT | TCCAGGAGTCCAAAGCCCACAA |
| *MdJMJ28* | GTGCTTCTTTGGAGGGCAGGTT | TTCTGTGGCGTTCTCGCTGTTG |
| *MdHDT03* | TGGAAAGAAGGGTGCCCACACT | TGATCGCCGCCAGCAGACTT |
| *MdHMA01* | GCTTCAGGCAGGCGATGGATAC | CGCAATGGACTCTCCGTCGTTC |
| *EF-1α* | ATTCAAGTATGCCTGGGTGC | CAGTCAGCCTGTGATGTTCC |
