# Supplementary material for: Identification and characterization of histone modification gene family reveal their critical responses to flower induction in apple
Source: BMC Plant Biol. 2018 Aug 20;18:173. doi: 10.1186/s12870-018-1388-0 (PMC6102887; doi:10.1186/s12870-018-1388-0)
Supplement: Supplementary file 4 — Table S4. Synteny analysis of MdHMs genes (DOCX 26 kb) [file 12870_2018_1388_MOESM4_ESM.docx]

Table S4. Synteny analysis of *MdHM* genes

| Region 1 | | | Region 2 | | | Syntenic genes | | | |
| --- | --- | --- | --- | --- | --- | --- | --- | --- | --- |
| Chr | Start | Stop | Chr | Start | Stop | Gene ID | Gene ID | Name | Name |
| Chr00 | 11221416 | 11227152 | Chr5 | 17697686 | 17702036 | MD00G1060700 | AT5G43990 | MdSDG02 | AtSDG18 |
| Chr00 | 19229525 | 19255727 | Chr5 | 2871365 | 2873789 | MD00G1091800 | AT5G09230 | MdSRT01 | AtSRT2 |
| Chr00 | 20399985 | 20405925 | Chr4 | 11008558 | 11013860 | MD00G1097500 | AT4G20400 | MdJMJ01 | AtJMJ14 |
| Chr01 | 2116071 | 2126575 | Chr4 | 16102595 | 16105550 | MD01G1005700 | AT4G33470 | MdHDA01 | AtHDA14 |
| Chr01 | 6225478 | 6230770 | Chr5 | 3038978 | 3041206 | MD01G1012000 | AT5G09790 | MdSDG05 | AtSDG15 |
| Chr01 | 18735301 | 18743567 | Chr3 | 22850811 | 22857016 | MD01G1080200 | AT3G61740 | MdSDG06 | AtSDG14 |
| Chr01 | 21501838 | 21506502 | Chr3 | 3229060 | 3232531 | MD01G1103000 | AT3G10390 | MdHDMA04 | AtHDMA1 |
| Chr01 | 21918410 | 21925954 | Chr5 | 1169153 | 1174992 | MD01G1106000 | AT5G04240 | MdJMJ04 | AtJMJ11 |
| Chr01 | 31068432 | 31075270 | Chr4 | 426847 | 431862 | MD01G1218500 | AT4G00990 | MdJMJ05 | AtJMJ27 |
| Chr01 | 31235382 | 31243106 | Chr5 | 21677146 | 21684122 | MD01G1220300 | AT5G53430 | MdSDG08 | AtSDG29 |
| Chr01 | 31551561 | 31553883 | Chr5 | 8295110 | 8297159 | MD01G1224300 | AT5G24330 | MdSDG09 | AtSDG34 |
| Chr02 | 2800117 | 2803571 | Chr2 | 8499000 | 8501294 | MD02G1037100 | AT2G19670 | MdPRMT01 | AtPRMT12 |
| Chr02 | 18671957 | 18677724 | Chr1 | 315983 | 319667 | MD02G1195100 | AT1G01920 | MdSDG12 | AtSDG42 |
| Chr02 | 32156564 | 32159136 | Chr1 | 27491184 | 27494182 | MD02G1267300 | AT1G73100 | MdSDG14 | AtSDG19 |
| Chr02 | 33429824 | 33438342 | Chr5 | 16953515 | 16961245 | MD02G1278400 | AT5G42400 | MdSDG15 | AtSDG25 |
| Chr03 | 17757824 | 17760240 | Chr1 | 2672198 | 2674721 | MD03G1154900 | AT1G08460 | MdHDA03 | AtHDA8 |
| Chr03 | 30449337 | 30452592 | Chr3 | 4478873 | 4481767 | MD03G1220300 | AT3G13682 | MdHDMA05 | AtHDMA2 |
| Chr03 | 37044296 | 37049505 | Chr4 | 886580 | 891987 | MD03G1294100 | AT4G02020 | MdSDG17 | AtSDG10 |
| Chr04 | 5512306 | 5533698 | Chr1 | 11846247 | 11856765 | MD04G1047300 | AT1G32750 | MdHAF01 | AtHAF1 |
| Chr04 | 31119965 | 31125573 | Chr3 | 1161366 | 1164977 | MD04G1231900 | AT3G04380 | MdSDG20 | AtSDG31 |
| Chr05 | 4535936 | 4538975 | Chr1 | 16308510 | 16311094 | MD05G1027900 | AT1G43245 | MdSDG21 | AtSDG41 |
| Chr05 | 5087165 | 5091475 | Chr1 | 28789698 | 28792719 | MD05G1031300 | AT1G76710 | MdSDG22 | AtSDG26 |
| Chr05 | 27696019 | 27702316 | Chr4 | 17896278 | 17899509 | MD05G1146000 | AT4G38130 | MdHDA05 | AtHDA19 |
| Chr05 | 46832063 | 46837369 | Chr1 | 4034479 | 4039065 | MD05G1351300 | AT1G11950 | MdJMJ09 | AtJMJ26 |
| Chr06 | 3210489 | 3217400 | Chr5 | 19046339 | 19051379 | MD06G1026100 | AT5G46910 | MdJMJ11 | AtJMJ13 |
| Chr06 | 30080831 | 30088073 | Chr3 | 17935256 | 17940792 | MD06G1159300 | AT3G48430 | MdJMJ12 | AtJMJ12 |
| Chr06 | 30764060 | 30767330 | Chr5 | 25315506 | 25318347 | MD06G1167500 | AT5G63110 | MdHDA08 | AtHDA6 |
| Chr08 | 412295 | 414742 | Chr1 | 23264486 | 23267221 | MD08G1004400 | AT1G62830 | MdHDMA09 | AtHDMA3 |
| Chr08 | 3272752 | 3276342 | Chr5 | 9099152 | 9101682 | MD08G1043300 | AT5G26040 | MdHDA11 | AtHDA2 |
| Chr08 | 12539643 | 12547230 | Chr4 | 15132011 | 15136696 | MD08G1132700 | AT4G31120 | MdPRMT02 | AtPRMT15 |
| Chr08 | 18359822 | 18371676 | Chr1 | 29039922 | 29049454 | MD08G1159600 | AT1G77300 | MdSDG28 | AtSDG8 |
| Chr08 | 23531368 | 23540505 | Chr1 | 29452555 | 29457373 | MD08G1186800 | AT1G78280 | MdJMJ16 | AtJMJ21 |
| Chr09 | 276859 | 283702 | Chr5 | 4501362 | 4506250 | MD09G1002600 | AT5G13960 | MdSDG29 | AtSDG33 |
| Chr09 | 7663156 | 7667217 | Chr3 | 7688434 | 7691567 | MD09G1105100 | AT3G21820 | MdSDG31 | AtSDG36 |
| Chr09 | 14189983 | 14200690 | Chr1 | 29716350 | 29724748 | MD09G1170000 | AT1G79000 | MdHAC02 | AtHAC1 |
| Chr10 | 23179136 | 23183061 | Chr4 | 17896278 | 17899509 | MD10G1145400 | AT4G38130 | MdHDA12 | AtHDA19 |
| Chr10 | 28954018 | 28965224 | Chr3 | 20213406 | 20217653 | MD10G1193500 | AT3G54610 | MdHAG28 | AtHAG1 |
| Chr10 | 39112395 | 39118408 | Chr4 | 11407804 | 11412279 | MD10G1304800 | AT4G21430 | MdJMJ17 | AtJMJ28 |
| Chr11 | 15067629 | 15068836 | Chr3 | 16297656 | 16299876 | MD11G1156600 | AT3G44750 | MdHDT04 | AtHDT1 |
| Chr11 | 15587775 | 15594185 | Chr3 | 16226519 | 16230009 | MD11G1159400 | AT3G44680 | MdHDA13 | AtHDA9 |
| Chr11 | 28737157 | 28751584 | Chr5 | 22567222 | 22571008 | MD11G1199100 | AT5G55760 | MdSRT03 | AtSRT1 |
| Chr11 | 39729728 | 39733774 | Chr2 | 9662638 | 9667038 | MD11G1279700 | AT2G22740 | MdSDG35 | AtSDG23 |
| Chr12 | 992429 | 994979 | Chr3 | 939900 | 942094 | MD12G1009500 | AT3G03750 | MdSDG36 | AtSDG20 |
| Chr12 | 5202290 | 5205217 | Chr5 | 2001071 | 2003112 | MD12G1046300 | AT5G06550 | MdJMJ21 | AtJMJ22 |
| Chr12 | 5900169 | 5904916 | Chr5 | 2033760 | 2036266 | MD12G1052100 | AT5G06620 | MdSDG38 | AtSDG38 |
| Chr12 | 29418485 | 29426130 | Chr1 | 2921064 | 2925826 | MD12G1216600 | AT1G09060 | MdJMJ22 | AtJMJ24 |
| Chr12 | 31721067 | 31728936 | Chr1 | 2736656 | 2743881 | MD12G1246900 | AT1G08620 | MdJMJ23 | AtJMJ16 |
| Chr13 | 4859546 | 4861180 | Chr1 | 9248152 | 9249943 | MD13G1069000 | AT1G26760 | MdSDG42 | AtSDG35 |
| Chr13 | 10396202 | 10397436 | Chr2 | 8491192 | 8492920 | MD13G1134900 | AT2G19640 | MdSDG44 | AtSDG39 |
| Chr13 | 13630857 | 13636161 | Chr5 | 19871191 | 19874974 | MD13G1168500 | AT5G49020 | MdPRMT03 | AtPRMT14 |
| Chr13 | 39189832 | 39205478 | Chr1 | 1754130 | 1761909 | MD13G1279000 | AT1G05830 | MdSDG46 | AtSDG30 |
| Chr14 | 1296467 | 1300281 | Chr5 | 981859 | 984289 | MD14G1014900 | AT5G03740 | MdHDT07 | AtHDT3 |
| Chr14 | 15999174 | 16014561 | Chr1 | 23544554 | 23553232 | MD14G1103700 | AT1G63490 | MdJMJ24 | AtJMJ17 |
| Chr14 | 25770292 | 25774371 | Chr5 | 20480626 | 20484945 | MD14G1163700 | AT5G50320 | MdHAG40 | AtHAG3 |
| Chr14 | 25922669 | 25930130 | Chr3 | 17935256 | 17940792 | MD14G1165600 | AT3G48430 | MdJMJ25 | AtJMJ12 |
| Chr14 | 26919946 | 26923646 | Chr5 | 25299933 | 25302476 | MD14G1175900 | AT5G63080 | MdJMJ26 | AtJMJ20 |
| Chr14 | 29675771 | 29680612 | Chr5 | 24566783 | 24571072 | MD14G1211400 | AT5G61060 | MdHDA15 | AtHDA5 |
| Chr14 | 30389133 | 30394179 | Chr3 | 6361159 | 6365705 | MD14G1222300 | AT3G18520 | MdHDA16 | AtHDA15 |
| Chr15 | 952195 | 960029 | Chr4 | 9217988 | 9226280 | MD15G1016200 | AT4G16310 | MdHDMA15 | AtHDMA4 |
| Chr15 | 9701529 | 9713384 | Chr1 | 29039922 | 29049454 | MD15G1133700 | AT1G77300 | MdSDG49 | AtSDG8 |
| Chr15 | 10452526 | 10458584 | Chr2 | 18258569 | 18261542 | MD15G1141800 | AT2G44150 | MdSDG50 | AtSDG7 |
| Chr15 | 16122666 | 16125915 | Chr5 | 22952767 | 22955739 | MD15G1202500 | AT5G56740 | MdHAG43 | AtHAG2 |
| Chr15 | 23660726 | 23673807 | Chr2 | 10097265 | 10103398 | MD15G1271600 | AT2G23740 | MdSDG51 | AtSDG6 |
| Chr15 | 26076132 | 26083928 | Chr2 | 9955092 | 9960386 | MD15G1285900 | AT2G23380 | MdSDG52 | AtSDG1 |
| Chr15 | 42986658 | 42991004 | Chr2 | 7773220 | 7776825 | MD15G1356600 | AT2G17900 | MdSDG54 | AtSDG37 |
| Chr15 | 43242221 | 43247915 | Chr5 | 25828160 | 25830669 | MD15G1358600 | AT5G64610 | MdHAM02 | AtHAM1 |
| Chr16 | 4767410 | 4769836 | Chr1 | 4805306 | 4807517 | MD16G1067900 | AT1G14030 | MdSDG56 | AtSDG43 |
| Chr16 | 23360135 | 23367316 | Chr4 | 7823894 | 7827004 | MD16G1228800 | AT4G13460 | MdSDG59 | AtSDG22 |
| Chr16 | 31350911 | 31359039 | Chr4 | 15024444 | 15027672 | MD16G1258900 | AT4G30860 | MdSDG60 | AtSDG4 |
| Chr17 | 7563670 | 7574912 | Chr4 | 8651406 | 8662587 | MD17G1091000 | AT4G15180 | MdSDG62 | AtSDG2 |
| Chr17 | 15124029 | 15128657 | Chr1 | 29716350 | 29724748 | MD17G1157200 | AT1G79000 | MdHAC04 | AtHAC1 |
| Chr17 | 34663025 | 34667892 | Chr5 | 5666823 | 5668907 | MD17G1287300 | AT5G17240 | MdSDG64 | AtSDG40 |
