# Supplementary material for: Identification and characterization of histone modification gene family reveal their critical responses to flower induction in apple
Source: BMC Plant Biol. 2018 Aug 20;18:173. doi: 10.1186/s12870-018-1388-0 (PMC6102887; doi:10.1186/s12870-018-1388-0)
Supplement: Supplementary file 5 — Table S5. Synteny information of MdHMs and AtHMs genes (DOCX 25 kb) [file 12870_2018_1388_MOESM5_ESM.docx]

**Table S5.** Synteny information of *MdHM* and *AtHM* genes

| Region 1 | | | Region 2 | | | Syntenic genes | | | |
| --- | --- | --- | --- | --- | --- | --- | --- | --- | --- |
| Chr | Start | Stop | Chr | Start | Stop | Gene ID | Gene ID | Name | Name |
| Chr00 | 11221416 | 11227152 | Chr02 | 31984152 | 31988373 | MD00G1060700 | MD02G1265700 | MdSDG02 | MdSDG13 |
| Chr00 | 13391674 | 13396040 | Chr12 | 17778780 | 17783537 | MD00G1068300 | MD12G1112200 | MdSDG03 | MdSDG39 |
| Chr00 | 20399985 | 20405925 | Chr10 | 33658731 | 33664694 | MD00G1097500 | MD10G1241100 | MdJMJ01 | MdJMJ19 |
| Chr00 | 42102840 | 42107157 | Chr03 | 37044296 | 37049505 | MD00G1179500 | MD03G1294100 | MdSDG04 | MdSDG17 |
| Chr01 | 6225478 | 6230770 | Chr15 | 38110853 | 38121027 | MD01G1012000 | MD15G1338000 | MdSDG05 | MdSDG53 |
| Chr01 | 21918410 | 21925954 | Chr07 | 24961209 | 24968468 | MD01G1106000 | MD07G1172400 | MdJMJ04 | MdJMJ15 |
| Chr01 | 22117920 | 22120431 | Chr07 | 25218471 | 25220575 | MD01G1108300 | MD07G1174400 | MdHAG02 | MdHAG20 |
| Chr01 | 31235382 | 31243106 | Chr07 | 35044515 | 35050998 | MD01G1220300 | MD07G1289800 | MdSDG08 | MdSDG27 |
| Chr01 | 32421522 | 32424967 | Chr07 | 36481233 | 36485077 | MD01G1237100 | MD07G1309700 | MdHAG03 | MdHAG22 |
| Chr02 | 2800117 | 2803571 | Chr15 | 13877192 | 13880754 | MD02G1037100 | MD15G1177300 | MdPRMT01 | MdPRMT07 |
| Chr02 | 5889116 | 5892401 | Chr15 | 16122666 | 16125915 | MD02G1072700 | MD15G1202500 | MdHAG04 | MdHAG43 |
| Chr02 | 7209065 | 7213147 | Chr15 | 17506951 | 17511056 | MD02G1091000 | MD15G1217100 | MdHAG05 | MdHAG44 |
| Chr02 | 13109967 | 13120994 | Chr15 | 23660726 | 23673807 | MD02G1157000 | MD15G1271600 | MdSDG10 | MdSDG51 |
| Chr02 | 16534385 | 16536020 | Chr15 | 27619139 | 27620785 | MD02G1183200 | MD15G1295100 | MdHAG06 | MdHAG45 |
| Chr02 | 17023746 | 17030795 | Chr15 | 28108459 | 28113279 | MD02G1187100 | MD15G1298200 | MdHAG07 | MdHAG46 |
| Chr02 | 32156564 | 32159136 | Chr07 | 4759934 | 4762952 | MD02G1267300 | MD07G1051900 | MdSDG14 | MdSDG24 |
| Chr02 | 35513057 | 35516059 | Chr07 | 1943940 | 1947016 | MD02G1300400 | MD07G1023300 | MdHAG08 | MdHAG19 |
| Chr03 | 5142593 | 5144741 | Chr11 | 5848192 | 5849680 | MD03G1064100 | MD11G1067900 | MdHAG10 | MdHAG29 |
| Chr03 | 13404889 | 13407175 | Chr11 | 15065356 | 15067627 | MD03G1134300 | MD11G1156500 | MdHDT01 | MdHDT03 |
| Chr03 | 13407177 | 13408268 | Chr11 | 15067629 | 15068836 | MD03G1134400 | MD11G1156600 | MdHDT02 | MdHDT04 |
| Chr03 | 13915110 | 13921648 | Chr11 | 15587775 | 15594185 | MD03G1137300 | MD11G1159400 | MdHDA02 | MdHDA13 |
| Chr03 | 24650309 | 24660667 | Chr11 | 28737157 | 28751584 | MD03G1179400 | MD11G1199100 | MdSRT02 | MdSRT03 |
| Chr03 | 34908001 | 34910443 | Chr11 | 40215468 | 40218157 | MD03G1263400 | MD11G1284200 | MdHAG11 | MdHAG30 |
| Chr04 | 3226153 | 3233332 | Chr13 | 39189832 | 39205478 | MD04G1028500 | MD13G1279000 | MdSDG18 | MdSDG46 |
| Chr04 | 5512306 | 5533698 | Chr10 | 28954018 | 28965224 | MD04G1047300 | MD10G1193500 | MdHAF01 | MdHAG28 |
| Chr04 | 6175213 | 6185872 | Chr11 | 39729728 | 39733774 | MD04G1052400 | MD11G1279700 | MdSDG19 | MdSDG35 |
| Chr04 | 26843628 | 26844492 | Chr12 | 27427126 | 27429716 | MD04G1177300 | MD12G1192400 | MdHAG12 | MdHAG32 |
| Chr04 | 28862118 | 28866548 | Chr12 | 29418485 | 29426130 | MD04G1202800 | MD12G1216600 | MdJMJ06 | MdJMJ22 |
| Chr04 | 30023580 | 30024086 | Chr12 | 30969553 | 30970155 | MD04G1217600 | MD12G1234800 | MdHAG13 | MdHAG33 |
| Chr04 | 30997445 | 31004908 | Chr12 | 31721067 | 31728936 | MD04G1229800 | MD12G1246900 | MdJMJ07 | MdJMJ23 |
| Chr04 | 31119965 | 31125573 | Chr12 | 31925657 | 31927317 | MD04G1231900 | MD12G1250000 | MdSDG20 | MdSDG40 |
| Chr05 | 5087165 | 5091475 | Chr10 | 4256109 | 4263816 | MD05G1031300 | MD10G1032900 | MdSDG22 | MdSDG33 |
| Chr05 | 7054677 | 7054979 | Chr09 | 260743 | 261795 | MD05G1042000 | MD09G1002300 | MdHAG14 | MdHAG24 |
| Chr05 | 12764381 | 12769823 | Chr10 | 10999013 | 11004377 | MD05G1067900 | MD10G1077800 | MdHDMA06 | MdHDMA11 |
| Chr05 | 27696019 | 27702316 | Chr10 | 23179136 | 23183061 | MD05G1146000 | MD10G1145400 | MdHDA05 | MdHDA12 |
| Chr05 | 37715068 | 37716473 | Chr10 | 32344668 | 32349000 | MD05G1244800 | MD10G1226200 | MdSDG23 | MdSDG34 |
| Chr05 | 45286027 | 45292287 | Chr10 | 39112395 | 39118408 | MD05G1326700 | MD10G1304800 | MdJMJ08 | MdJMJ17 |
| Chr05 | 46375447 | 46377318 | Chr10 | 40189290 | 40193258 | MD05G1344100 | MD10G1320100 | MdHDMA07 | MdHDMA12 |
| Chr05 | 46832063 | 46837369 | Chr10 | 40607236 | 40611894 | MD05G1351300 | MD10G1325700 | MdJMJ09 | MdJMJ20 |
| Chr06 | 3210489 | 3217400 | Chr16 | 38164869 | 38171753 | MD06G1026100 | MD16G1280000 | MdJMJ11 | MdJMJ28 |
| Chr06 | 5201301 | 5202452 | Chr14 | 2068097 | 2069926 | MD06G1040400 | MD14G1023000 | MdHAG15 | MdHAG39 |
| Chr06 | 20068477 | 20088135 | Chr14 | 15999174 | 16014561 | MD06G1081900 | MD14G1103700 | MdJMJ13 | MdJMJ24 |
| Chr06 | 28285227 | 28288186 | Chr14 | 24671843 | 24674689 | MD06G1137800 | MD14G1152600 | MdHDMA08 | MdHDMA13 |
| Chr06 | 30080831 | 30088073 | Chr14 | 25922669 | 25930130 | MD06G1159300 | MD14G1165600 | MdJMJ12 | MdJMJ25 |
| Chr06 | 30764060 | 30767330 | Chr14 | 26710479 | 26713749 | MD06G1167500 | MD14G1173000 | MdHDA08 | MdHDA14 |
| Chr06 | 33552172 | 33561400 | Chr14 | 29675771 | 29680612 | MD06G1202300 | MD14G1211400 | MdHDA09 | MdHDA15 |
| Chr06 | 34443691 | 34449271 | Chr14 | 30389133 | 30394179 | MD06G1211800 | MD14G1222300 | MdHDA10 | MdHDA16 |
| Chr06 | 36524997 | 36528018 | Chr14 | 31967643 | 31970243 | MD06G1234100 | MD14G1240800 | MdHAG16 | MdHAG41 |
| Chr08 | 412295 | 414742 | Chr15 | 233872 | 236319 | MD08G1004400 | MD15G1003800 | MdHDMA09 | MdHDMA14 |
| Chr08 | 1319733 | 1328087 | Chr15 | 952195 | 960029 | MD08G1017300 | MD15G1016200 | MdHDMA10 | MdHDMA15 |
| Chr08 | 12539643 | 12547230 | Chr15 | 7859494 | 7866979 | MD08G1132700 | MD15G1112100 | MdPRMT02 | MdPRMT05 |
| Chr08 | 13967368 | 13968363 | Chr15 | 8502403 | 8504958 | MD08G1142000 | MD15G1118400 | MdHAG23 | MdHAG42 |
| Chr08 | 18359822 | 18371676 | Chr15 | 9701529 | 9713384 | MD08G1159600 | MD15G1133700 | MdSDG28 | MdSDG49 |
| Chr08 | 20910939 | 20917157 | Chr15 | 43242221 | 43247915 | MD08G1173300 | MD15G1358600 | MdHAM01 | MdHAM02 |
| Chr08 | 23531368 | 23540505 | Chr15 | 45518685 | 45527976 | MD08G1186800 | MD15G1372700 | MdJMJ16 | MdJMJ27 |
| Chr09 | 276859 | 283702 | Chr17 | 453801 | 461485 | MD09G1002600 | MD17G1006800 | MdSDG29 | MdSDG61 |
| Chr09 | 5797129 | 5803799 | Chr17 | 5879982 | 5889173 | MD09G1082800 | MD17G1073200 | MdHAC01 | MdHAC03 |
| Chr09 | 7552939 | 7564204 | Chr17 | 7563670 | 7574912 | MD09G1103200 | MD17G1091000 | MdSDG30 | MdSDG62 |
| Chr09 | 9986947 | 9993377 | Chr17 | 10261111 | 10265300 | MD09G1129500 | MD17G1118300 | MdSDG32 | MdSDG63 |
| Chr09 | 14189983 | 14200690 | Chr17 | 15124029 | 15128657 | MD09G1170000 | MD17G1157200 | MdHAC02 | MdHAC04 |
| Chr12 | 1657705 | 1659576 | Chr14 | 1296467 | 1300281 | MD12G1016900 | MD14G1014900 | MdHDT05 | MdHDT07 |
| Chr13 | 1306222 | 1309184 | Chr16 | 1406246 | 1409499 | MD13G1020900 | MD16G1019300 | MdSDG41 | MdSDG55 |
| Chr13 | 6171691 | 6173449 | Chr16 | 6088147 | 6089667 | MD13G1088000 | MD16G1088300 | MdHAG35 | MdHAG47 |
| Chr13 | 9861050 | 9866656 | Chr16 | 9508440 | 9514086 | MD13G1130100 | MD16G1130300 | MdSDG43 | MdSDG57 |
| Chr13 | 10396202 | 10397436 | Chr16 | 9999424 | 10002951 | MD13G1134900 | MD16G1130700 | MdSDG44 | MdSDG58 |
| Chr13 | 21848245 | 21853552 | Chr16 | 23360135 | 23367316 | MD13G1224000 | MD16G1228800 | MdSDG45 | MdSDG59 |
| Chr15 | 10452526 | 10458584 | Chr16 | 31350911 | 31359039 | MD15G1141800 | MD16G1258900 | MdSDG50 | MdSDG60 |
