# Supplementary figures and images for: Identification and characterization of histone modification gene family reveal their critical responses to flower induction in apple
Source: BMC Plant Biol. 2018 Aug 20;18:173. doi: 10.1186/s12870-018-1388-0 (PMC6102887; doi:10.1186/s12870-018-1388-0)

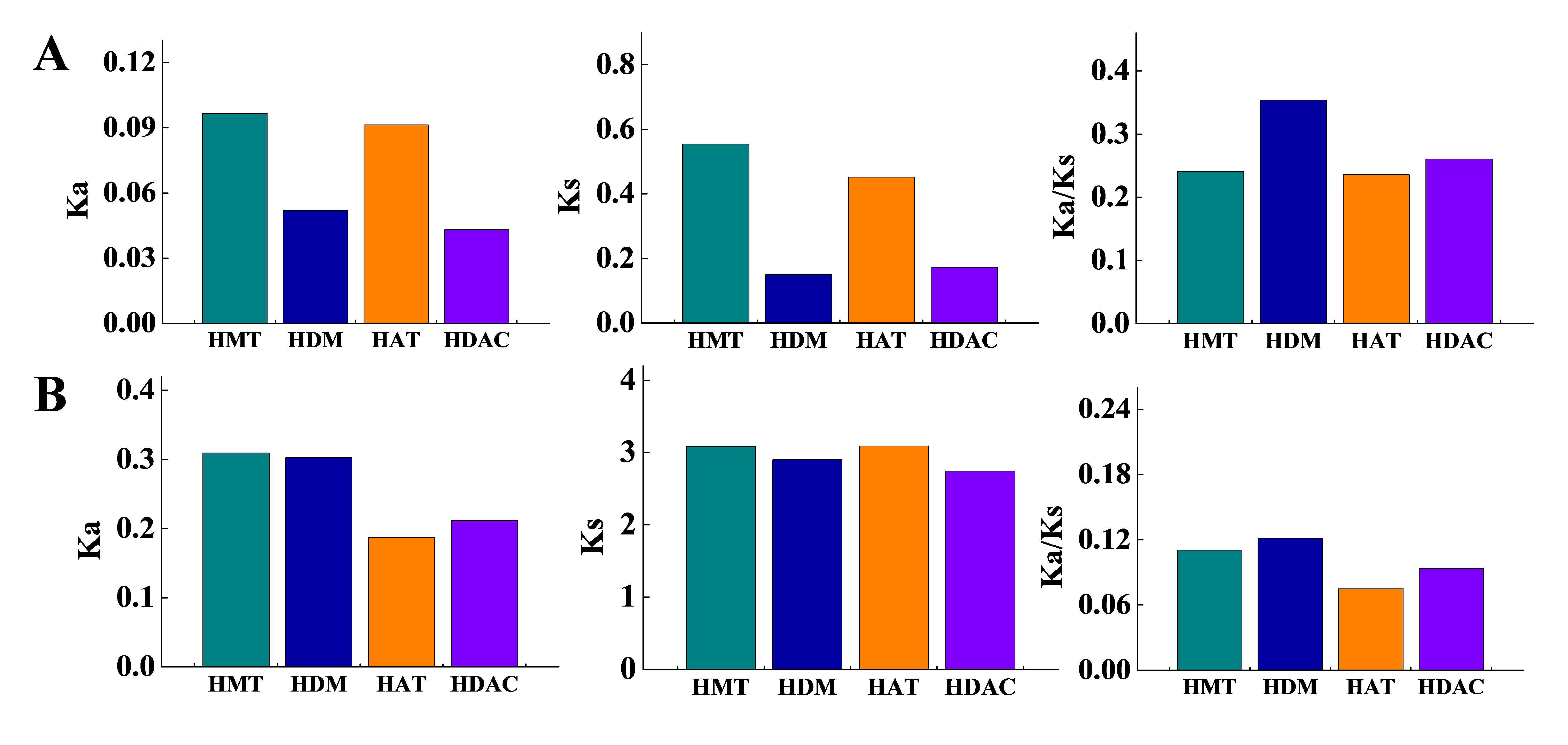

Supplement: Supplementary file 6 — Figure S1. Average Ka, Ks values of duplication gene pairs. (A) gene pairs of apple; (B) gene pairs of apple and Arabidopsis (TIF 367 kb) [file 12870_2018_1388_MOESM6_ESM.tif]

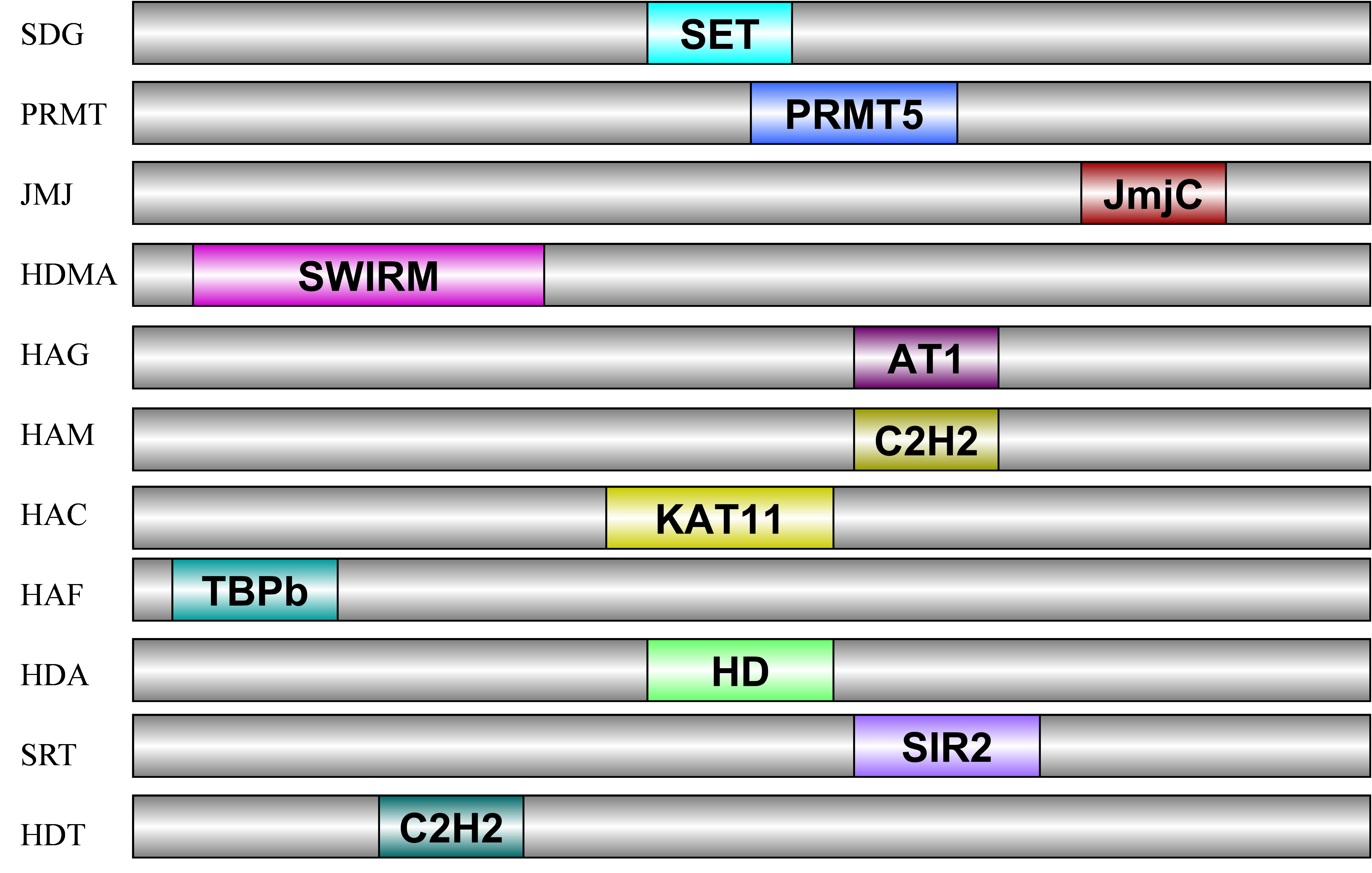

Supplement: Supplementary file 7 — Figure S2. Diagram of HMs typical conserved domains (TIF 846 kb) [file 12870_2018_1388_MOESM7_ESM.tif]

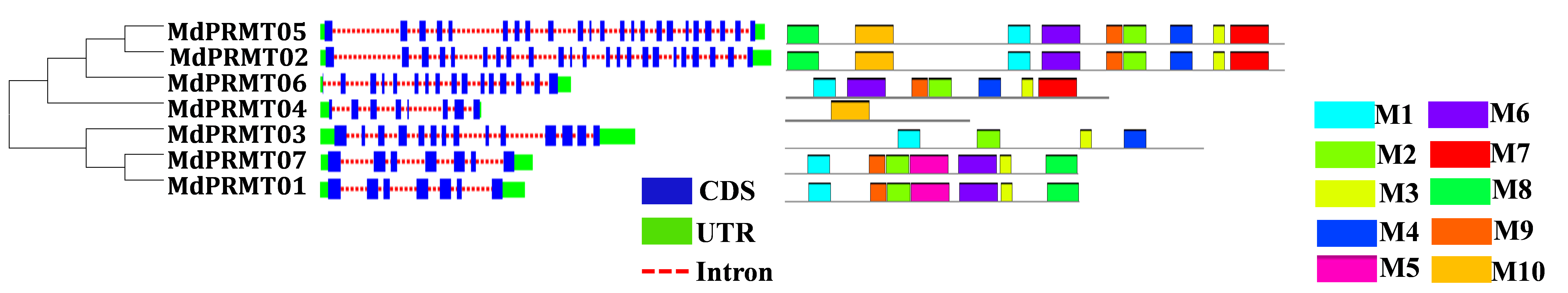

Supplement: Supplementary file 9 — Figure S4. Gene structure and protein motifs analysis of MdPRMTs gene family members (TIF 571 kb) [file 12870_2018_1388_MOESM9_ESM.tif]

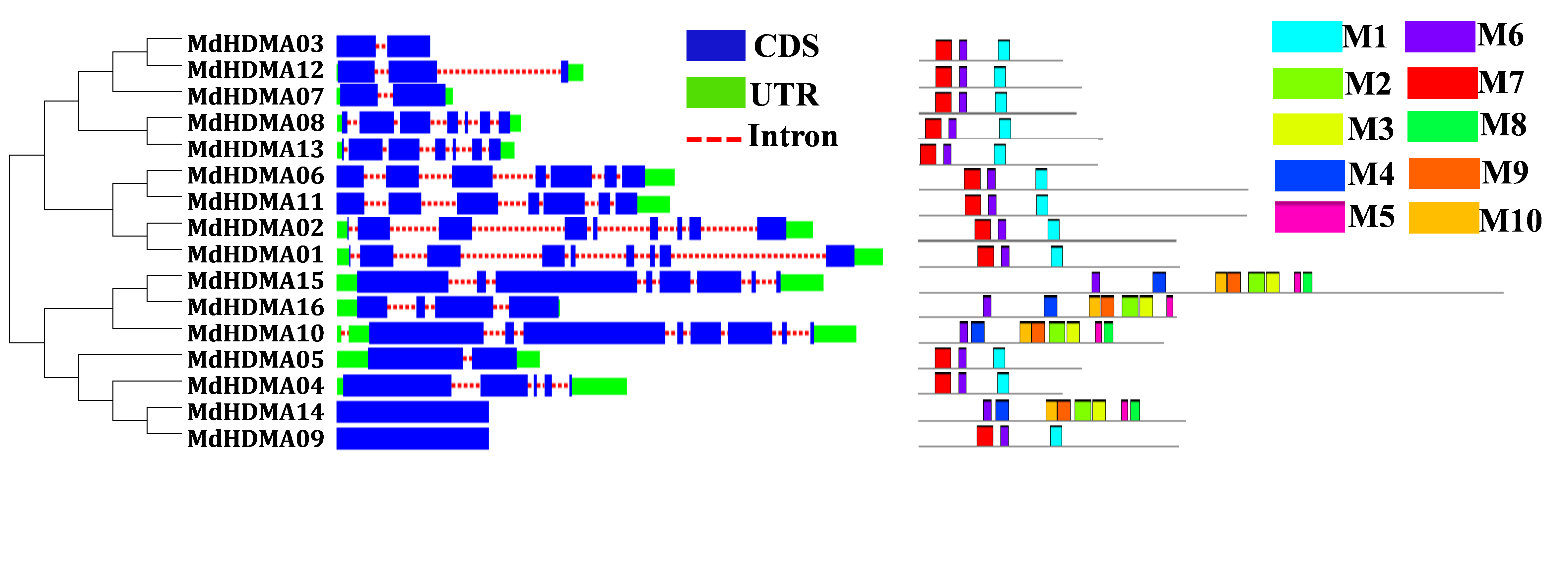

Supplement: Supplementary file 10 — Figure S5. Gene structure and protein motifs analysis of MdHDMAs gene family members (TIF 639 kb) [file 12870_2018_1388_MOESM10_ESM.tif]

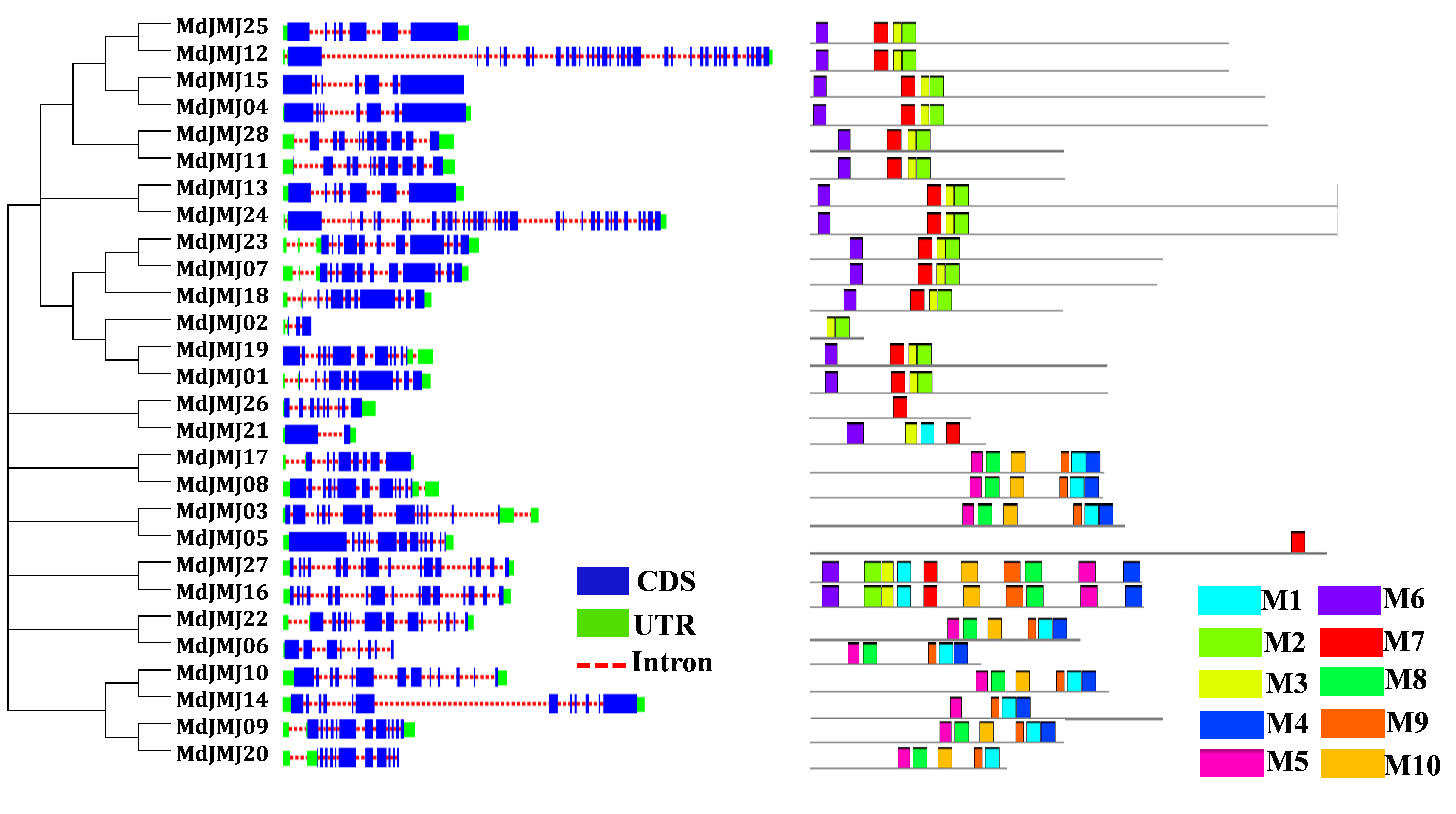

Supplement: Supplementary file 11 — Figure S6. Gene structure and protein motifs analysis of MdJMJs gene family members (TIF 1445 kb) [file 12870_2018_1388_MOESM11_ESM.tif]

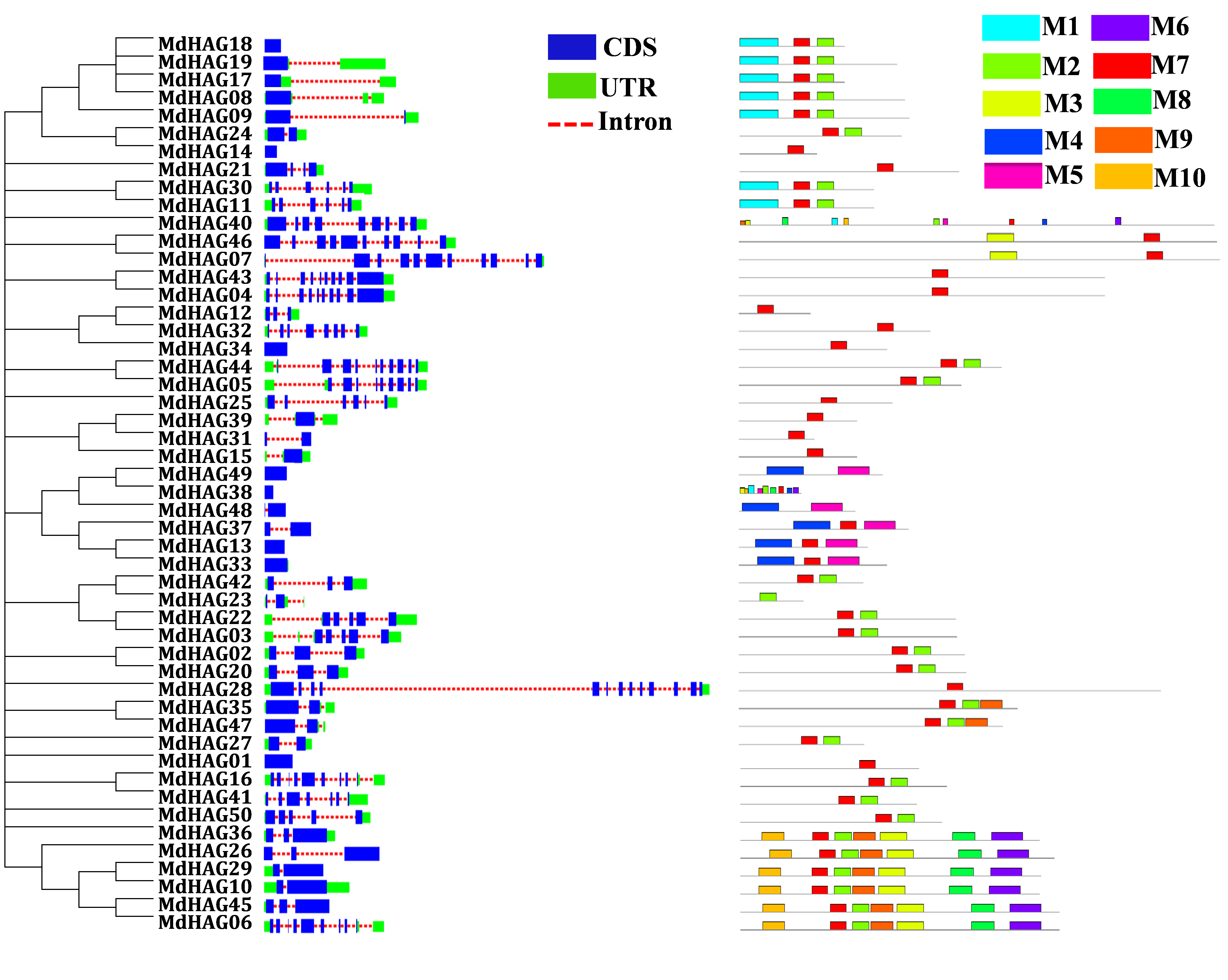

Supplement: Supplementary file 12 — Figure S7. Gene structure and protein motifs analysis of MdHAGs gene family members (TIF 1174 kb) [file 12870_2018_1388_MOESM12_ESM.tif]

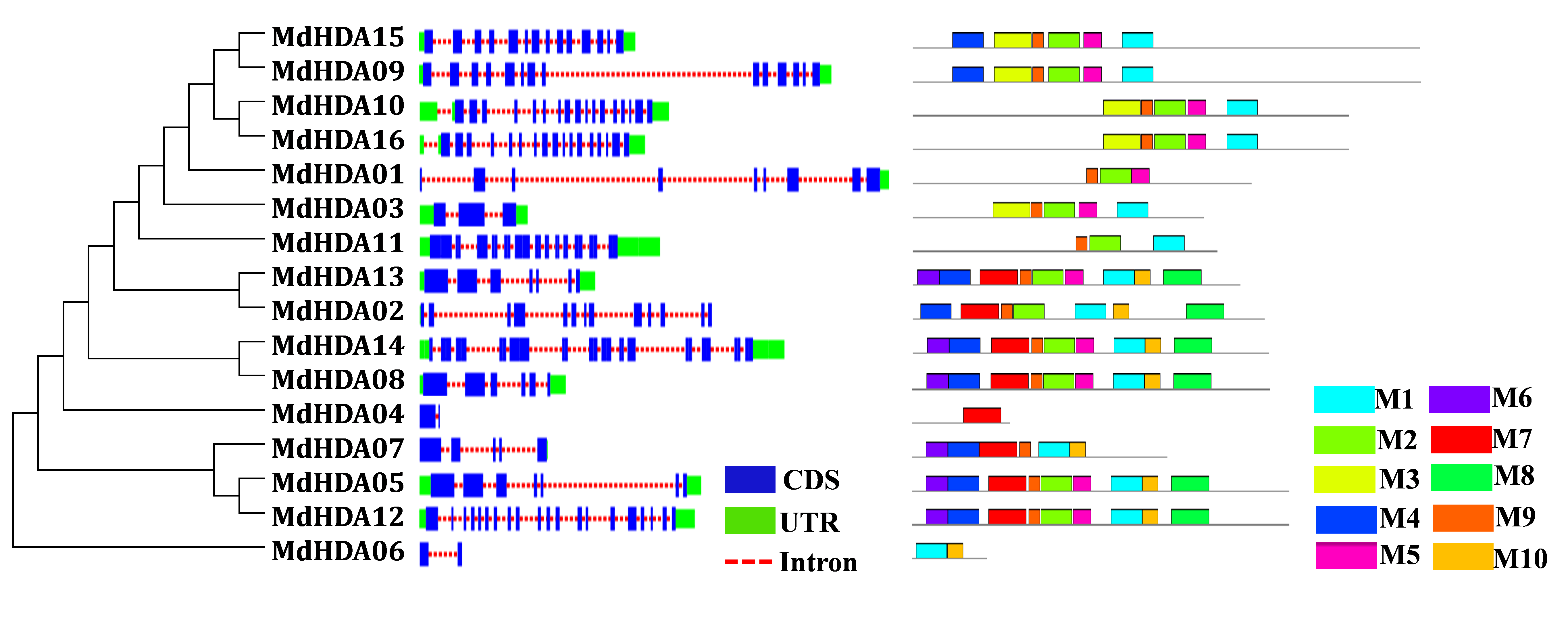

Supplement: Supplementary file 13 — Figure S8. Gene structure and protein motifs analysis of MdHDAs gene family members (TIF 1253 kb) [file 12870_2018_1388_MOESM13_ESM.tif]

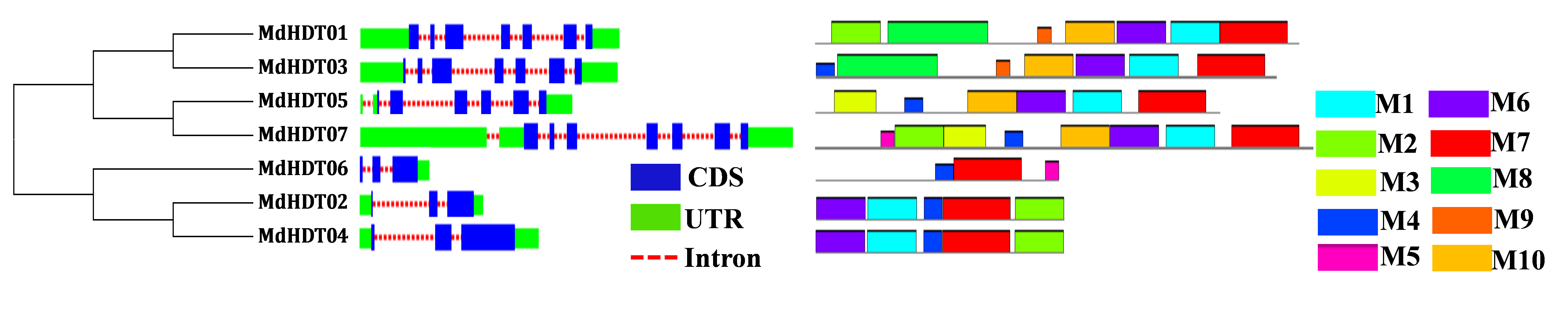

Supplement: Supplementary file 14 — Figure S9. Gene structure and protein motifs analysis of MdHDTs gene family members (TIF 429 kb) [file 12870_2018_1388_MOESM14_ESM.tif]

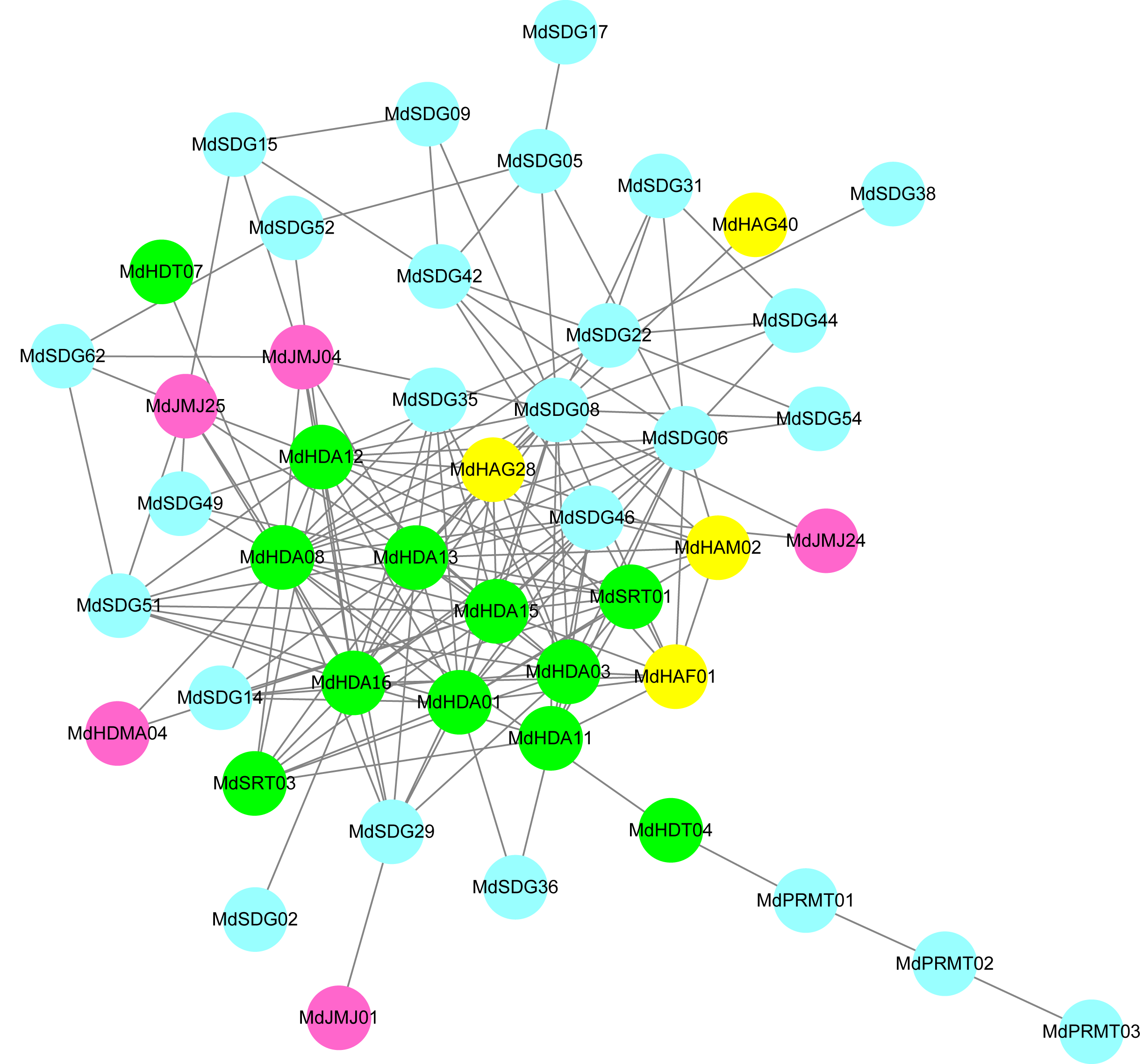

Supplement: Supplementary file 16 — Figure S10. Interaction networks analysis of MdHMs genes (TIF 1052 kb) [file 12870_2018_1388_MOESM16_ESM.tif]

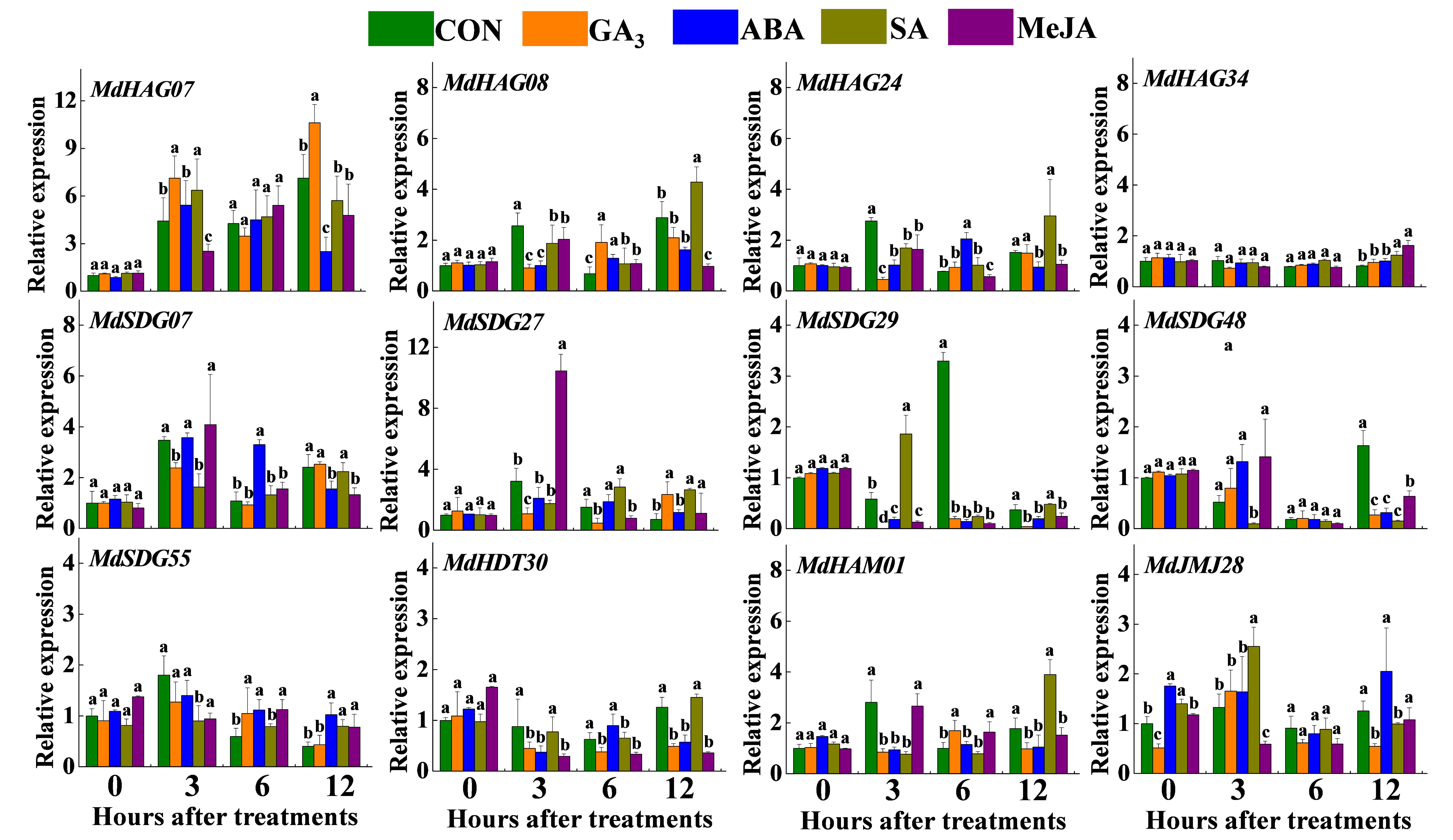

Supplement: Supplementary file 18 — Figure S11. Transcript levels of 12 MdHMs genes following GA3, ABA, SA, and MeJA in by qRT-PCR. Leaves were collected after 0, 3,6 and 12 h after treatment. Each value represents the mean ± standard error of three replicates. Means followed by small letters are significantly different at the 0.05 level (the same below). (TIF 3532 kb) [file 12870_2018_1388_MOESM18_ESM.tif]

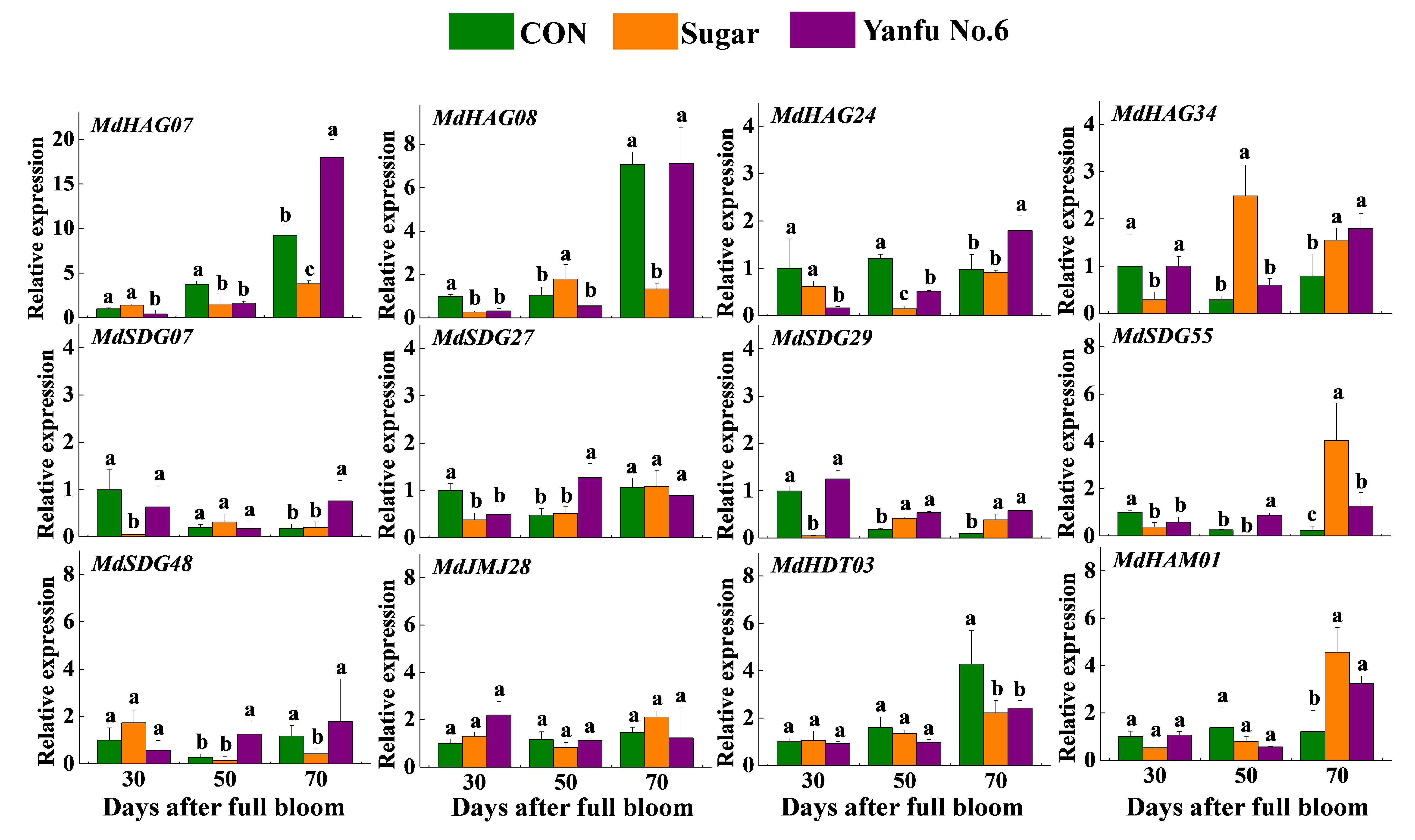

Supplement: Supplementary file 19 — Figure S12. Transcript levels of 12 MdHMs genes following sugar treatment and in Yanfu No.6. Terminal buds were collected from 30, 50, and 70 DAFB. (TIF 734 kb) [file 12870_2018_1388_MOESM19_ESM.tif]
