# Supplementary material for: Identification and characterization of histone modification gene family reveal their critical responses to flower induction in apple
Source: BMC Plant Biol. 2018 Aug 20;18:173. doi: 10.1186/s12870-018-1388-0 (PMC6102887; doi:10.1186/s12870-018-1388-0)
Supplement: Supplementary file 15 — Table S6. Motif sequences of MdHMs proteins (DOCX 16 kb) [file 12870_2018_1388_MOESM15_ESM.docx]

Table S6. Motif sequences of MdHM proteins

| SDG | Motif 1 | GNIARFINHSCEPNL |
| --- | --- | --- |
|  | Motif 2 | GWGLRSWDSIRAGAFICEYAG |
|  | Motif 3 | NCSCIQKNGGDFPYTSNGILV |
|  | Motif 4 | STEEEPLALSIVSSGGYEDSVEDSNVLIYSGQGGN |
|  | Motif 5 | EVADQKLERGNLALQRSLHHGNEVRVIRG |
|  | Motif 6 | GAVPGVEVGDIFFFRMELCLVGLHAPTMGGIDYM |
|  | Motif 7 | HIMFFAAEDIPPMQELTYHYN |
|  | Motif 8 | LVYECGPSCKCPPSCYNRVSQ |
|  | Motif 9 | PDGKVKKMFCHCGATGCRKRL |
|  | Motif 10 | NPTGKVYVYDGLYKIHESWVDKGKSGCNVFKYKLVRLPGQP |
| PRMT | Motif 1 | LRLYLDYVGYLYQRMDPLPEQERLEIGYR |
|  | Motif 2 | PEKADILVSELLGSFGDNELSPECLDGAQR |
|  | Motif 3 | SAMIHGFAGYFDATL |
|  | Motif 4 | HKDIAHFETAYVVKLHNIARLAPPQPVFT |
|  | Motif 5 | WLKPTGVMYPSHATMWVAPIRTGLGDQKNNDYDSSMDDWERFTDETKNYY |
|  | Motif 6 | AQTYETFEKDTMKYIQYQRAICRALQDRVPDDKASSVTTVLMVVGAGRGP |
|  | Motif 7 | IEPSTSTPNMFSWFPIFFPLRTPITLSPGASLEVHFWRCCSPTKVWYEWC |
|  | Motif 8 | NGSFSMKRSKENHRLMEVEFSCEIRQQSGELVPRFTNRYFI |
|  | Motif 9 | VKLEGWENIVTIISCDMRYWD |
|  | Motif 10 | WIDLDSEDEILRCDSETTLKQEIAWASHLSLQACLLPAPKGKSCANYARC |
| HDMA | Motif 1 | IHVPSYSSWFSPDHIHHCEVRFLPEFFDSRSPSKNPSLYKYYRNTIVAQS |
|  | Motif 2 | GSVRRVFDFLEAWGLINYTPSAPNK |
|  | Motif 3 | MGSGWADKDTLHLLEALMHYGDDWRKVAQHVGRSEK |
|  | Motif 4 | KKIIVIGAGPAGLTAARHLQRQGFSVTILEARSRIGGRVYT |
|  | Motif 5 | ERMLLDWHLANLEYANASLMSNLSMAYWDQDDPYE |
|  | Motif 6 | MGGDHCFIPGGNETFVRSLAEGLPIFYERTVQSIRYGSDGV |
|  | Motif 7 | AILITVPLGCLKAETIKFSPPLPHWKHSSILRLGFGVLNKVVLEFPDVFW |
|  | Motif 8 | DYFGATAEETELRGQCFMFWNVKKTVGAPVLIALVVGKAAI |
|  | Motif 9 | YGAYSYVAVGASGEDYDILGR |
|  | Motif 10 | FFAGEATNKQYPATMHGAFLSGMREAANI |
| JMJ | Motif 1 | IHPIHDQSFYLTLEHKRRLKEEFGVEPWTFVQRLGEAVFIPAGCPHQVRN |
|  | Motif 2 | QNAGEFVLTFPRAYHSGFNCGFNCAEAVNVAPVDWLPHGQIAIELYQEQG |
|  | Motif 3 | EEQPDLLHKLVTQLSPSILKSEGVPVYRC |
|  | Motif 4 | QSCIKVALDFVSPESLEECLRLTEEFRLLPKNHRAKEDKLEVKKMTLYAV |
|  | Motif 5 | DEIEHFQRHWINGEPVIVRNVLDKTSGLSWEPMVMWRAFRE |
|  | Motif 6 | IDEAPVFYPTIEEFEDTLGYIAKIRPLAESYGICRIVPPPSWTP |
|  | Motif 7 | DISGVLVPWLYIGMCFSSFCWHVEDHHLYSLNYMHWGAPKLWYGIPGSDA |
|  | Motif 8 | VKALDCFDWCEVEINIHQFFMGYLEGRLHRSGWPEMLKLKDWPSSTLFEE |
|  | Motif 9 | GGALWDIFRRQDVPKLQEYLRKHFKEFRH |
|  | Motif 10 | KPDMGPKTYIAYGFLEELGRGDSVTKLHCDMSDAVNILTHTAEVNLSNEQ |
| HAG | Motif 1 | MVCIRKATIDDLLAMQACNLFCLPENYQMKYYLYHILSWPQLLYVAEDYN |
|  | Motif 2 | VFAAEYVSLHVRKSNRAAFNLY |
|  | Motif 3 | VEEAEYLYRKCMENTEFFPHDIDKVLGNNLSLGTW |
|  | Motif 4 | WGTDEKVTVFCTWEPYASKEEALNFIKDVVVPHPWFRAICLDNRPIGA |
|  | Motif 5 | WTHLDRLEALVDVDNVGSQRLLEKVGFQREGVLRKYCMLKG |
|  | Motif 6 | NCKVIVTEVGGCDPVRHHIPHWKLLSCYEDLWCIKALKNED |
|  | Motif 7 | YILGLRVSPTHRRLGIGSKLV |
|  | Motif 8 | ALPWMQLPSVPDIFRPFGVHFLYGLGGEG |
|  | Motif 9 | KCGYVKFRTPTILVNPVYAHRVKVSSRVT |
|  | Motif 10 | RRCEVGPSGGLSLFTDLLGDPICRVRHSP |
| HDA | Motif 1 | PGAVVLQCGADSLSGDRLGCFNLSIKGHAECVRYMRSFNVP |
|  | Motif 2 | ELLKVHRRVLYVDIDVHHGDGVEEAFYTTDRVMTVSFHKFG |
|  | Motif 3 | TPDTYANEHSARAARLAAGLCADLAKAIVSGRAKNGFALVRPPGHHAGV |
|  | Motif 4 | ALLAHYGLLQNMQVLKPYPARDRDLCRFHADDYVAFLRNIT |
|  | Motif 5 | FFPGTGHIKDVGAGTGKNYALNVP |
|  | Motif 6 | VSYFYDPEVGNYYYGQGHPMKPHRIRMT |
|  | Motif 7 | RFNVGEDCPVFDGLYSFCQTYAGGSVGGAVKLNHGICDISINWAGGLHHA |
|  | Motif 8 | YYEYFGPDYTLHVAPSNMENKNSHMLLEEIRSKLLENLSRLQHAPSVQFQ |
|  | Motif 9 | EASGFCYVNDIVLAI |
|  | Motif 10 | LLLLGGGGYTIRNVARCWCYE |
| HDT | Motif 1 | KKGAHTATPHPAKKGGKTPAT |
|  | Motif 2 | VHFCGYQTCLAEYPFNRLCLC |
|  | Motif 3 | VNVTEVAKKPIKSEQKEA |
|  | Motif 4 | VGNQKLVL |
|  | Motif 5 | LGYPFC |
|  | Motif 6 | AESTKTPANDKKPKFVTPEKT |
|  | Motif 7 | APKSSGAFHCQPCNRSFNSDGALQSHTKA |
|  | Motif 8 | EEDLPLNFTGNGNIVEAKPAPPKTNTVKPESSGKQKVKIEEPI |
|  | Motif 9 | EDMLGA |
|  | Motif 10 | DDDSEEDEETPKKADVGKKRP |
