# Supplementary material for: Identification and characterization of histone modification gene family reveal their critical responses to flower induction in apple
Source: BMC Plant Biol. 2018 Aug 20;18:173. doi: 10.1186/s12870-018-1388-0 (PMC6102887; doi:10.1186/s12870-018-1388-0)
Supplement: Supplementary file 20 — Table S8. Summary of HMs in different species (DOCX 13 kb) [file 12870_2018_1388_MOESM20_ESM.docx]

Table S8. Summary of HMs in different species

| Types | | *Malus*  *domestica* | *Solanum lycopersicum* | *Citrus*  *sinensis* | *Aradopsis thaliana* | *Oryza sativa* | *Zea mays* |
| --- | --- | --- | --- | --- | --- | --- | --- |
| HMT | Total | 71 | 52 | 47 | 78 | 42 | 43 |
|  | Density* | 0.110 | 0.068 | 0.128 | 0.577 | 0.113 | 0.018 |
| HDM | Total | 44 | 26 | 23 | 24 | 24 | 14 |
|  | Density | 0.068 | 0.034 | 0.062 | 0.177 | 0.064 | 0.006 |
| HAT | Total | 57 | 32 | 50 | 12 | 8 | 12 |
|  | Density | 0.088 | 0.042 | 0.136 | 0.088 | 0.021 | 0.005 |
| HDAC | Total | 26 | 14 | 16 | 18 | 18 | 16 |
|  | Density | 0.040 | 0.018 | 0.043 | 0.133 | 0.048 | 0.006 |
| HM | Total | 198 | 124 | 136 | 132 | 92 | 85 |
|  | Density | 0.307 | 0.163 | 0.370 | 0.755 | 0.247 | 0.037 |

*Density: Number / Mb.
